# Astrocytic Ketogenesis Protects Central Nervous System Injury via CPT1A Stabilization and Epigenetic Regulation

**DOI:** 10.1002/advs.78393

**Published:** 2026-10-20

**Authors:** Dishui Pan, Haoming Shu, Rui Gao, Yilong Ren, Wu Ye, Xuhui Ge, Yao Gu, Yufeng Zhu, Xiaokun Wang, Honglin Teng, Xuhui Zhou, Wei Liu, Yang Gao, Jianquan Zhao, Weihua Cai

**Affiliations:** ^1^ Department of Orthopedics The First Affiliated Hospital of Nanjing Medical University Nanjing Jiangsu China; ^2^ Department of Orthopedics Shanghai General Hospital Shanghai Jiao Tong University School of Medicine Shanghai China; ^3^ Department of Orthopedics Second Affiliated Hospital of Naval Medical University Shanghai China; ^4^ Department of Stress Medicine Faculty of Psychology Naval Medical University Shanghai China; ^5^ Department of Orthopedics (Spine Surgery) The First Affiliated Hospital of Wenzhou Medical University Wenzhou Zhejiang China; ^6^ Department of Orthopedics Fourth Medical Centre of Chinese PLA General Hospital Beijing China

**Keywords:** astrocytes, β‐OHB, CPT1A, CNS injury, epigenetics, ketogenesis

## Abstract

Following central nervous system (CNS) injury, reactive astrocytes proliferate and encapsulate the lesion to facilitate repair. However, the specific metabolic characteristics remain unknown. Here, through integrated single‐cell and spatial transcriptomics, we uncovered a subset of astrocytes that undergo enhanced lipid and ketone metabolic reprogramming following spinal cord injury (SCI). The ketone body β‐hydroxybutyrate (β‐OHB) inhibited autophagic degradation of carnitine‐palmitoyltransferase‐1A (CPT1A) via P300‐mediated lysine β‐hydroxybutyrylation (Kbhb). Astrocyte‐specific deletion of *Cpt1a* led to impaired wound healing, reduced axonal sparing and worsened locomotor recovery after SCI, effects rescued by exogenous β‐OHB. Mechanistically, ketogenesis promotes wound repair and lipid metabolic reprogramming, forming a positive feedback loop via H3K9bhb‐dependent epigenetic remodeling in astrocytes after SCI. Furthermore, ketogenic diet (KD) promoted tissue repair across multiple CNS injury models. Clinically, elevated β‐OHB levels were associated with better tissue preservation and functional recovery in SCI patients. Collectively, our findings underscore the critical role of astrocytic lipid metabolic and epigenetic reprogramming after CNS injury and identify KD as a promising therapeutic strategy.

## Graphical abstract

A distinct astrocyte subpopulation, enriched at the lesion border, drives CNS repair through enhanced ketone body metabolism. This process promotes neuronal survival and axonal sparing via CPT1A stabilization and H3K9bhb‐dependent epigenetic reprogramming, positioning astrocytic ketogenesis as a promising therapeutic target for tissue regeneration.

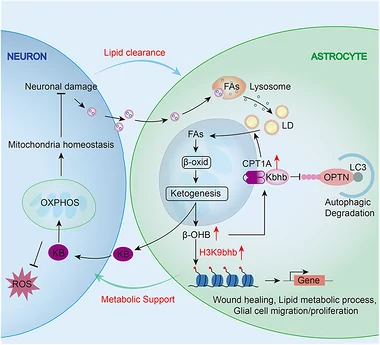

## 1 Introduction

Injuries to the central nervous system (CNS), primarily traumatic brain/spinal cord injury (TBI/SCI) and stroke, represent leading causes of mortality and permanent disability in motor, sensory, and autonomic functions [1, 2]. The development of effective therapies has been hampered by a critical gap in understanding the underlying mechanisms of CNS damage and repair. Astrocytes, the most abundant glial cells in the CNS, play multifaceted roles in both health and disease. In the healthy adult CNS, these cells are largely quiescent, but following injury, they undergo a transformation known as astrocyte reactivity [3, 4]. This process involves profound molecular, structural, and functional alterations. Triggered by significant tissue damage, astrocyte reactivity leads to the formation of protective borders around areas affected by trauma, ischemia, infection, or neurodegeneration, achieved through a comprehensive remodeling of their morphological, genomic, metabolic, and functional characteristics [5, 6, 7]. Historically, the prevailing view was that these astrocytic borders impeded regeneration [8, 9, 10]. Emerging evidence, however, reveals that newly generated astrocytes at the lesion site help contain inflammation, construct protective barriers, and promote tissue repair and functional recovery after traumatic CNS injuries [11, 12, 13]. Despite the growing recognition of the importance of these proliferated, border‐forming astrocytes, their fundamental properties, particularly their metabolic reprogramming and how it relates to their transcriptional and functional heterogeneity remain poorly defined.

Astrocytes, which are intimately associated with blood vessels and functionally integrated with all other CNS cell types, serve as a critical conduit for metabolite transport. Their end feet ensheathe nearly all capillaries in the CNS, enabling the uptake of nutrients such as glucose from the blood, its conversion to lactate within astrocytes, and subsequent shuttling to energetically demanding neurons [14, 15]. We and others have demonstrated that astrocyte‐derived lactate sustains neuronal function in various neurological disorders [16, 17, 18, 19]. An analogous transport system operates in reverse, allowing toxic metabolites and metabolic byproducts from neurons and other CNS cells to be transferred back through astrocytes for breakdown or recycling [15, 20, 21]. Thus, astrocytes are pivotal to CNS metabolic homeostasis, both by nourishing neurons and clearing waste. Recent advances in integrative multimodal sequencing have begun to reveal the remarkable heterogeneity of astrocytes, including neuroinflammatory, neurotoxic reactive, and disease‐associated subtypes, and underscore the regulatory importance of metabolism in these populations [22, 23, 24, 25]. Given the high lipid content of the CNS, precise fatty acid regulation is essential for homeostasis. Astrocytes can absorb lipids from overactive neurons to shield them from lipotoxicity, resulting in significant lipid accumulation within astrocytes and subsequent reprogramming of lipid metabolism [21, 26]. An identified subtype of reactive astrocytes is characterized by high lipid content and a neurotoxic phenotype [27]. However, it remains unknown whether abnormal lipid metabolism occurs in reactive astrocytes following CNS injury, and if so, how it influences astrocytic function and disease progression.

Therefore, this study seeks to elucidate the cellular and metabolic heterogeneity of reactive astrocytes, with a specific focus on lipid metabolism, to identify potential targets for modulating disease progression. By integrating single‐cell RNA sequencing (scRNA‐seq) with spatial transcriptomics (ST), we identified a distinct astrocyte subpopulation characterized by enhanced fatty acid and ketone body (KB) metabolism that is markedly enriched at the lesion border. These astrocytes facilitate neuronal survival, axonal sparing, and wound repair through carnitine‐palmitoyltransferase‐1A (CPT1A) protein stabilization and H3K9bhb‐dependent epigenetic reprogramming. Collectively, our findings underscore the critical role of metabolic and epigenetic remodeling in astrocytes following CNS injury and position astrocytic ketogenesis as a promising therapeutic target for enhancing CNS tissue repair.

## 2 Results

### 2.1 Astrocytic Lipid and Ketone Metabolism is Upregulated after SCI

To elucidate the astrocytic metabolic shifts after SCI, we reanalyzed a recently published snRNA‐seq dataset (GSE234774). Cell populations were annotated based on the expression of specific marker genes (Figure 1A and Figure S1A). Subsequent sub‐clustering analysis of the astrocyte population identified five distinct subpopulations: lipid metabolism‐associated (cluster 0), neuron‐associated (cluster 1), cell migration‐associated (cluster 2), immune response‐associated (cluster 3), and cell differentiation‐related (cluster 4) (Figure 1B and Figure S1B). Gene expression analysis revealed a significant upregulation of lipid metabolism‐related genes in cluster 0, and further KEGG enrichment analysis also identified significantly upregulated lipid metabolism pathways (Figure 1C and Figure S1C). To further assess the metabolic states, we utilized gene set scoring, which confirmed that fatty acid metabolism was significantly enriched in cluster 0 following SCI (Figure 1D). Time‐series analysis demonstrated that both fatty acid and ketone body metabolism in astrocytes were disrupted immediately post‐SCI but underwent a gradual recovery, peaking at 14‐day post‐injury (dpi; Figure 1E,F). GSEA confirmed these findings, showing a significant enhancement of these pathways at 14 dpi (Figure 1G). To investigate the spatial distribution of these metabolic changes after SCI, we reanalyzed a corresponding ST dataset (GSE234774). Analysis of the isolated astrocyte population revealed high expression of lipid metabolism‐related genes after SCI (Figure S1D,E). Spatial gene set scoring further demonstrated that fatty acid and ketone body metabolism in astrocytes were significantly enhanced in the perilesional areas (Figure 1H). Interestingly, additional gene set scoring for astrocyte proliferation and wound healing revealed that astrocytes with enriched fatty acid and ketone body metabolism showed marked spatial overlap with astrocytes associated with proliferation and wound healing (Figure S1F). These findings were validated through the reanalysis of an independent scRNA‐seq dataset (GSE205029), which showed a similar expansion of a lipid metabolism‐enriched astrocyte subpopulation following injury (Figure S2A–H), with the activities of fatty acid metabolism and ketone body metabolism peaking at 14 dpi (Figure S2I–K).

**FIGURE 1:**
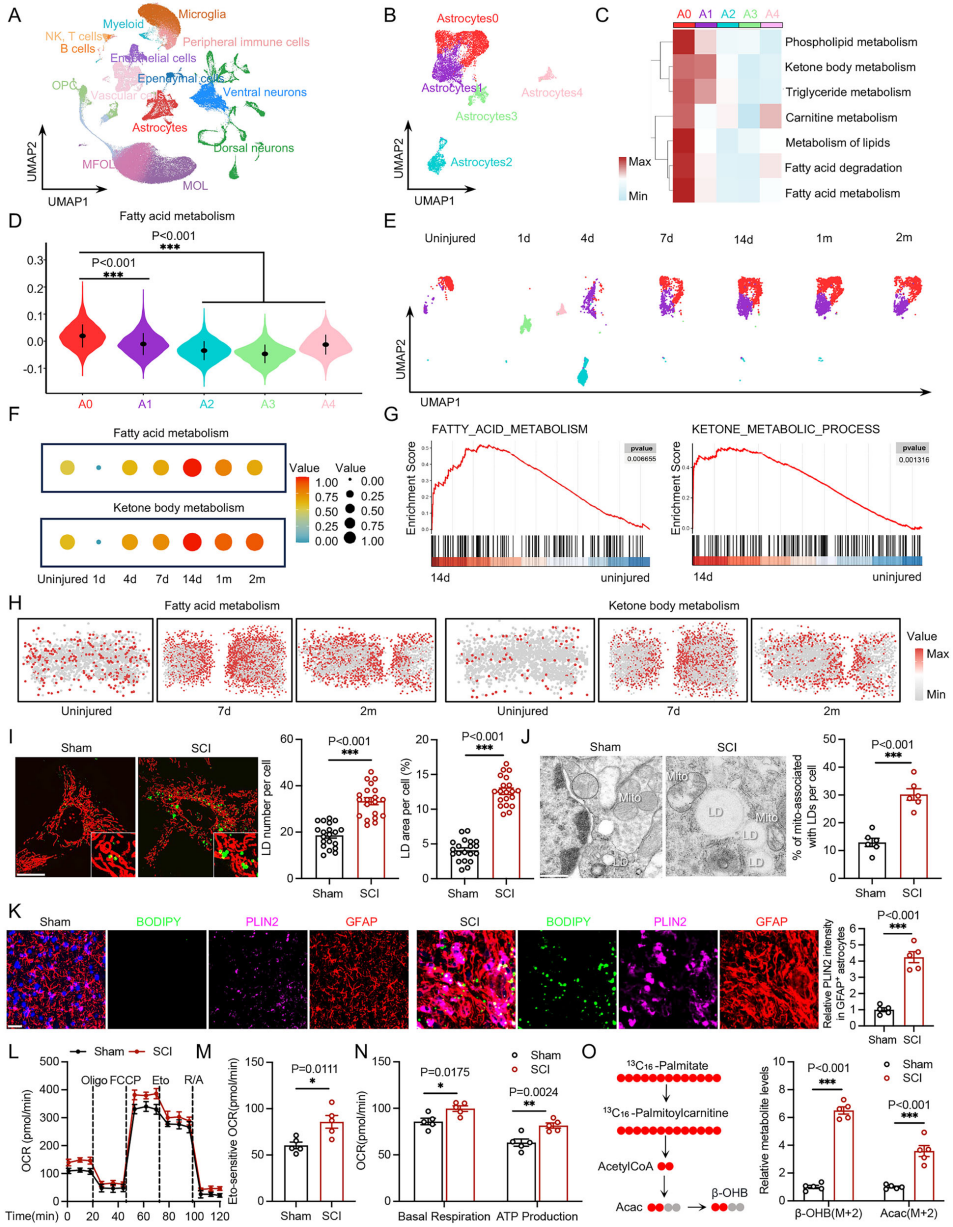
Astrocytic lipid and ketone metabolism is upregulated after SCI. (A) UMAP plot showing clusters of spinal cord cells after SCI. (B) UMAP plot showing populations of re‐clustered astrocytes. (C) KEGG enrichment analysis of lipid metabolism‐related pathways in astrocyte subclusters. (D) Gene set scoring of fatty acid metabolism in astrocyte subclusters. (E) UMAP plots of astrocyte subclusters at the indicated times. (F) Dot plot shows the fatty acid and ketone body metabolism in astrocytes at the indicated time points. (G) GSEA plots of fatty acid and ketone body metabolism pathways in astrocytes at 14 dpi. (H) Spatial distribution of gene set scores for fatty acid and ketone body metabolism across spatial barcodes. (I) Representative images of BODIPY and MitoTracker co‐staining in sorted sham or SCI astrocytes (*n* = 20 per group). Scale bar = 20 µm. (J) Representative TEM images of astrocytes sorted from mouse spinal cord tissues (*n* = 6 per group). Scale bar = 1 µm. (K) Representative images of PLIN2, BODIPY, and GFAP co‐staining in the spinal cord of sham and SCI mice at 14 dpi (*n* = 5 per group). Scale bar = 20 µm. (L‐N) OCR analysis was performed on astrocytes isolated from the sham and SCI groups (L), with statistical quantification of Etomoxir‐sensitive oxygen consumption (M), basal respiration and ATP production (N) (*n* = 5 per group). (O) Schematic diagram showing the flux of the ^13^C_16_‐Palmitate tracer in astrocytes. Grey represents ^12^C atoms, and red represents ^13^C atoms. Bar plot showing the relative abundance of metabolites in astrocytes (*n* = 5 per group). Data were analyzed using two‐tailed unpaired Student's *t*‐test (I–K, M‐O) and one‐way ANOVA followed by post hoc Bonferroni correction (D).

To further investigate the metabolic alterations in astrocytes, we isolated astrocytes from sham‐operated or injured spinal cords using magnet‐activated cell sorting (MACS; Figure S3A). Lipid droplet (LD) staining revealed significant LD accumulation in astrocytes after SCI (Figure 1I). Transmission electron microscopy (TEM) confirmed substantial LD accumulation in astrocytes post‐SCI, with mitochondria clustered around LD (Figure 1J). Immunofluorescence demonstrated an increased population of LD‐bearing astrocytes surrounding the injury site, paralleled by elevated expression of the LD‐associated protein PLIN2 (Figure 1K). Furthermore, perilesional astrocytes with high PLIN2 expression exhibited enhanced proliferative capacity marked by SOX9^+^ (Figure S3B). Cellular metabolic analysis revealed significant metabolic reprogramming in these astrocytes, characterized by enhanced fatty acid oxidation (FAO; Figure 1L,M). This efficient FAO process generated acetyl‐CoA derivatives, which enhanced basal respiration and ATP production (Figure 1N), while also serving as substrates for ketogenesis. Metabolic flux analysis indicated that this reprogramming significantly promoted the production of β‐OHB (Figure 1O), accompanied by increased expression of the key ketogenic enzymes HMGCL and HMGCS2 in astrocytes post‐injury (Figure S3C). Crucially, the transfer of conditioned medium from astrocytes post‐injury significantly supplied energy to neurons subjected to oxygen‐glucose deprivation/reoxygenation (OGD/R) (Figure S3D,E). This intervention also mitigated oxidative stress, maintained mitochondrial homeostasis, and enhanced neuronal survival (Figure S3F–J). Simultaneously, we observed that astrocytes with enhanced lipid metabolism following injury exhibited superior proliferative and wound‐healing capacities (Figure S3K,L). However, these beneficial effects were reversed by the administration of the ketogenesis inhibitor Hymeglusin (Hym; Figure S3E–L). Furthermore, exogenous supplementation of β‐OHB significantly improved neuronal survival and suppressed oxidative stress (Figure S3M–R). Notably, the addition of β‐OHB to astrocytes promoted proliferation and accelerated wound healing (Figure S3S,T). Collectively, these data demonstrate that after SCI, the upregulation of lipid and ketone metabolism in astrocytes not only exerts neuroprotective effects but also promotes astrocytic proliferation and wound healing through ketone metabolism.

### 2.2 β‐OHB Promotes CPT1A Kbhb Modification at Lysine 344 via P300

Notably, astrocytes activated by lipid metabolism exhibit a structural coupling between lipid droplets and mitochondria (Figure 1I,J). Studies have shown that PLIN2‐CPT1A may function as a key component of a lipid droplet‐mitochondria anchoring complex, recruiting adipose triglyceride lipase to the anchoring region to initiate lipolysis, thereby enhancing FAO [28]. We observed an elevation of CPT1A in the spinal cord post‐injury, reaching a peak at 14 dpi (Figure S4A). Western blot analysis further confirmed the upregulation of CPT1A protein within astrocytes (Figure S4B). Multiple studies have reported that β‐OHB can induce protein lysine Kbhb modification [29, 30, 31]. To assess the status of protein Kbhb after SCI, we first measured the levels of β‐OHB in spinal cord tissues. The results showed that both β‐OHB content and global Kbhb levels were significantly increased after SCI (Figure 2A,B). Subsequently, we used immunoprecipitation‐mass spectrometry to investigate potential Kbhb‐modified proteins and, interestingly, identified CPT1A as a Kbhb‐modified protein in astrocytes (Figure 2C). Co‐IP experiments revealed an increase in Kbhb modification of CPT1A after injury or β‐OHB addition, and reciprocal Co‐IP further confirmed CPT1A as a substrate for Kbhb (Figure 2D,E; Figure S4C,D). To identify the transferase responsible for CPT1A‐Kbhb modification, we transfected various histone acetyltransferases into primary astrocytes. Immunoprecipitation analysis demonstrated that P300 specifically promoted the formation of CPT1A‐Kbhb (Figure 2F). To characterize the involvement of P300, we silenced *p300* in astrocytes (Figure S4E) and found that *p300* knockdown reduced CPT1A Kbhb levels (Figure 2G). Further validation in primary astrocytes transfected with HA‐P300 demonstrated that the overexpression of P300 significantly promoted the β‐OHB‐induced increase in CPT1A Kbhb levels (Figure S4F). The application of dose‐dependent C646 (a specific P300 inhibitor) in astrocytes gradually reduced Kbhb levels (Figure S4G). The inhibitory effect on CPT1A Kbhb was further validated following C646 treatment in astrocytes (Figure S4H). Notably, after silencing *p300* or blocking CPT1A Kbhb modification with C646, the enhanced astrocytic proliferation and wound healing by β‐OHB was partially reversed (Figure S4I,J).

**FIGURE 2:**
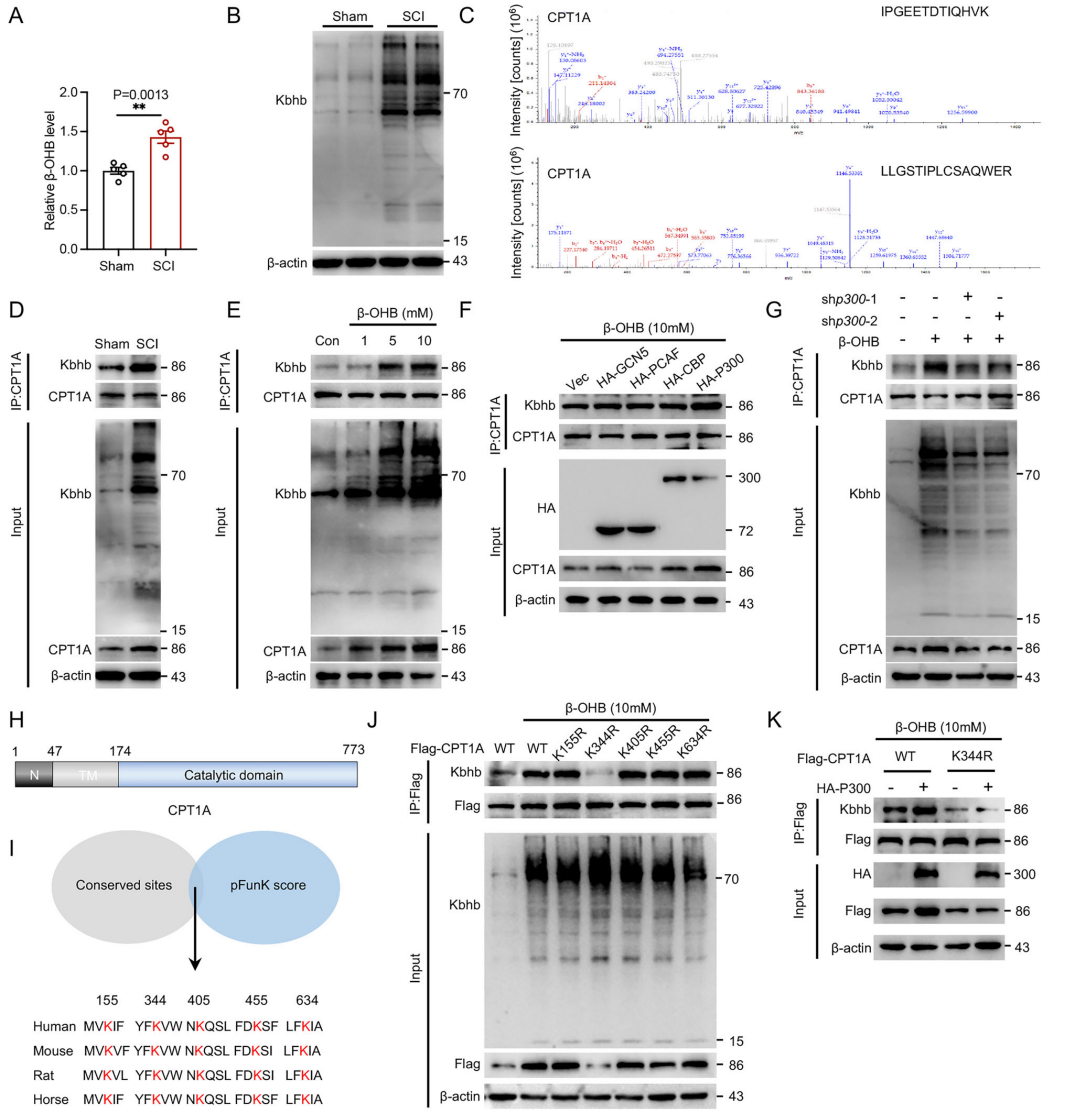
β‐OHB promotes CPT1A Kbhb modification at lysine 344 via P300. (A) Relative levels of spinal cord β‐OHB in sham and SCI mice at 14 dpi (*n* = 5 per group). (B) Kbhb analysis of spinal cord tissues from sham and SCI mice at 14 dpi. (C) The specific CPT1A peptide segments were identified in the Kbhb complex obtained by immunoprecipitation from astrocytes. (D) CPT1A was immunoprecipitated from spinal cord tissues under specific conditions, and its Kbhb modification level was detected using a Kbhb antibody. (E) Primary astrocytes treated with a concentration gradient of β‐OHB were subjected to immunoprecipitation with a CPT1A antibody, followed by detection of CPT1A Kbhb modification levels using a Kbhb antibody. (F) Primary astrocytes were transfected with either an empty vector or constructing HA‐GCN5, HA‐PCAF, HA‐CBP, or HA‐P300. The Kbhb modification was further analyzed. (G) Astrocytes transfected with or without sh*p300* were treated with 10 mM β‐OHB for 24 h. Cell lysates were then subjected to immunoprecipitation with an anti‐CPT1A antibody, followed by detecting CPT1A Kbhb levels. (H) Schematic diagram of the CPT1A molecular structure. (I) Schematic diagram of the lysine mutation sites in CPT1A protein. (J) HEK293T cells transfected with Flag‐CPT1A (WT or mutants) were treated with 10 mM β‐OHB for 24 h, followed by immunoprecipitation and immunoblotting to detect CPT1A Kbhb modification levels. (K) HEK293T cells co‐transfected with Flag‐CPT1A (WT or K344R) and HA‐P300, followed by immunoprecipitation with a Flag antibody and immunoblotting to detect the Kbhb modification levels of CPT1A. Data were analyzed using a two‐tailed unpaired Student's *t*‐test (A).

Structural analysis shows that CPT1A consists of a short, conserved N‐terminal regulatory domain and a long C‐terminal catalytic domain, connected by two transmembrane segments spanning the outer mitochondrial membrane (Figure 2H). To precisely pinpoint the specific lysine residues undergoing Kbhb modification in CPT1A, we screened potential lysine sites based on pFunK scores [32] and multi‐species alignment and constructed a series of wild‐type (WT) and point‐mutated CPT1A plasmids (Figure 2I). In cells transfected with WT, K155R, K344R, K405R, K455R, or K634R and treated with 10 mM β‐OHB for 24 h, a selective reduction in the Kbhb signal was detected only in the K344R mutant (Figure 2J). Consistently, the CPT1A K344R mutation abolished P300‐mediated enhancement of CPT1A Kbhb (Figure 2K). These results showed that CPT1A underwent P300‐mediated Kbhb modification at K344.

### 2.3 Kbhb Inhibits CPT1A Selective Autophagic Degradation via the Cargo Receptor OPTN

Having shown that CPT1A undergoes Kbhb modification, we further investigated the regulatory effects of this modification on CPT1A. Experimental results showed that CPT1A protein abundance increased with β‐OHB concentration in a dose‐dependent manner, while treatment with C646 blocked this effect (Figure 3A). Notably, *Cpt1a* mRNA levels remained unchanged during this process (Figure 3A). Similarly, *p300* knockdown led to decreased CPT1A protein levels without affecting mRNA expression (Figure 3B), indicating that Kbhb regulates CPT1A levels not at the transcriptional level but rather at the posttranslational level. Under conditions of protein synthesis inhibition by cycloheximide (CHX), *p300* knockdown significantly promoted the degradation of CPT1A protein (Figure 3C). Correspondingly, the addition of β‐OHB maintained CPT1A protein stability, whereas the C646 abolished this stabilization (Figure 3D). Importantly, transfection of Flag‐CPT1A K344R also abolished the stabilization effect induced by β‐OHB treatment (Figure S5A). Given that P300 can also mediate protein acetylation (Kac) and that astrocytes possess sufficient acetyl‐CoA substrates during metabolic reprogramming [33], we further investigated the regulatory role of Kac on CPT1A. The results showed that although CPT1A underwent P300‐mediated Kac modification, the level of acetylation did not affect the protein stability of CPT1A (Figure S5B–D).

**FIGURE 3:**
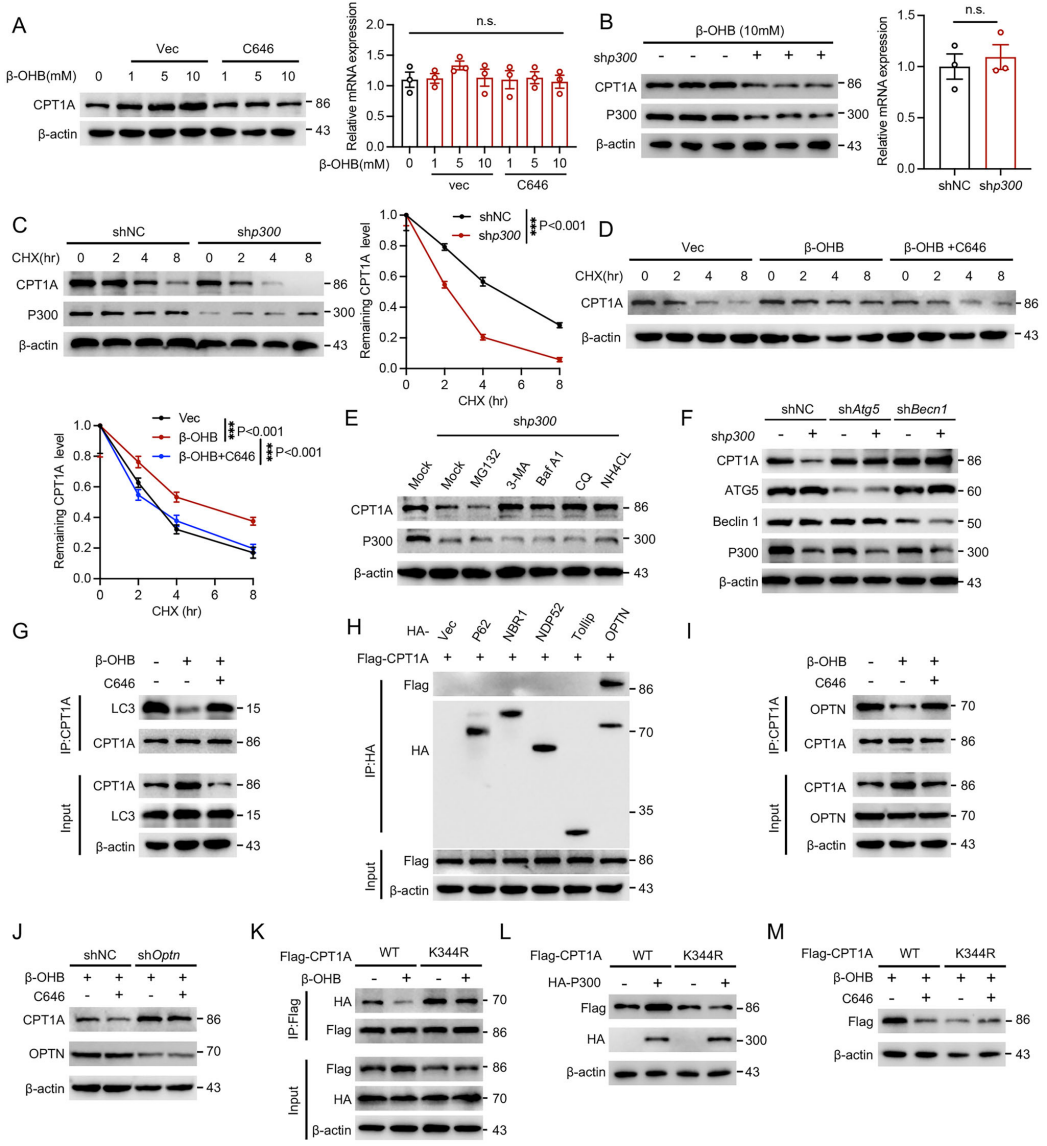
Kbhb inhibits CPT1A selective autophagic degradation via the cargo receptor OPTN. (A) Primary astrocytes were treated with different concentrations of β‐OHB in the presence or absence of C646, followed by immunoblot analysis of CPT1A protein levels and RT‐qPCR analysis of *Cpt1a* mRNA levels. (B) Primary astrocytes transfected with or without sh*p300* were treated with 10 mM β‐OHB for 24 h, followed by immunoblotting to assess CPT1A protein levels and RT‐qPCR analysis to determine *Cpt1a* mRNA expression. (C) Changes in CPT1A protein levels in primary astrocytes transfected with or without sh*p300* over the indicated time periods in the presence of cycloheximide (CHX, 10 µg mL^−1^). (D) Changes in CPT1A protein levels in primary astrocytes under specified treatment conditions over the indicated time periods in the presence of CHX. (E) CPT1A protein levels in primary astrocytes transfected with sh*p300* and treated with DMSO (Mock), MG132, 3‐MA, Baf A1, CQ, or NH4Cl. (F) Primary astrocytes were co‐transfected with either scramble shRNA or specific shRNAs (targeting *Atg5* or *Becn 1*) and either shNC or sh*p300*, followed by immunoblot analysis of the cell lysates. (G) Co‐IP analysis of CPT1A and LC3 in primary astrocytes with or without β‐OHB/C646 treatment. (H) Co‐IP analysis of the interaction between CPT1A and various cargo receptors in HEK293T cells. (I) Co‐IP analysis of CPT1A and OPTN in primary astrocytes with or without β‐OHB/C646 treatment. (J) Primary astrocytes transfected with or without sh*Optn* were treated with β‐OHB in the presence or absence of C646, followed by immunoblot analysis of cell lysates. (K) Co‐IP analysis of exogenous CPT1A and OPTN in HEK293T cells co‐transfected with Flag‐CPT1A (WT or K344R mutant) and HA‐OPTN with or without β‐OHB treatment. (L) HEK293T cells transfected with Flag‐CPT1A (WT or K344R mutant) were co‐transfected with or without HA‐P300, and cell lysates were analyzed by immunoblotting. (M) HEK293T cells transfected with Flag‐CPT1A (WT or K344R mutant) were treated with β‐OHB in the presence or absence of C646, and cell lysates were analyzed by immunoblotting. Data were analyzed using a two‐tailed unpaired Student's *t*‐test (B), one‐way ANOVA followed by post hoc Bonferroni correction (A), and two‐way ANOVA followed by post hoc Bonferroni correction (C,D).

Considering that the ubiquitin‐proteasome system and the autophagy‐lysosome pathway are the two major protein degradation mechanisms in cells, we evaluated CPT1A protein stability using various pharmacological treatments. We found that autophagy/lysosome inhibitors (including 3‐MA, Baf A1, chloroquine, and NH4Cl) significantly inhibited the protein degradation caused by the loss of CPT1A Kbhb, while the proteasome inhibitor MG132 had no such effect (Figure 3E). This suggests that CPT1A Kbhb modification primarily maintains protein stability by inhibiting the autophagy‐lysosome pathway. Autophagy is a tightly regulated process that depends on key regulators such as ATG5 and Beclin1, whose knockdown severely impairs autophagic function. Using astrocytes with *Atg5* or *Becn1* knockdown, we found that the CPT1A degradation induced by *p300* knockdown was significantly reversed (Figure 3F). This confirms that CPT1A Kbhb modification prevents protein degradation via the autophagy pathway. Furthermore, classical autophagy inducers such as rapamycin and EBSS led to CPT1A degradation in primary astrocytes (Figure S5E). Notably, rapamycin treatment abolished β‐OHB‐mediated CPT1A upregulation, while Baf A1 treatment reversed autophagy‐induced CPT1A degradation (Figure S5F,G). Importantly, β‐OHB treatment weakened the interaction between CPT1A and the autophagosome marker LC3, an effect that was blocked by C646 (Figure 3G). These results collectively demonstrate that CPT1A Kbhb modification maintains protein stability primarily by inhibiting the autophagic degradation pathway.

Recent studies have highlighted the crucial role of cargo receptors in the selective autophagic degradation of proteins [34, 35]. Our study identified a specific binding between CPT1A and the cargo receptor OPTN (Figure 3H). Elevated Kbhb levels reduced the interaction between CPT1A and OPTN, and this regulatory effect was abolished by C646 treatment (Figure 3I). Moreover, *Optn* knockdown significantly blocked the CPT1A degradation induced by Kbhb downregulation (Figure 3J). Overexpression of exogenous OPTN eliminated the stabilization of CPT1A mediated by Kbhb modification (Figure S5H). Notably, β‐OHB treatment failed to inhibit the interaction between the CPT1A K344R mutant and OPTN (Figure 3K). Correspondingly, P300 overexpression failed to promote the protein stability of CPT1A K344R (Figure 3L), and C646‐induced reduction of CPT1A was abolished when the K344 residue was mutated (Figure 3M). These data collectively demonstrate that Kbhb modification inhibits the autophagic degradation of CPT1A through the cargo receptor OPTN.

### 2.4 Astrocyte‐Specific Deletion of *Cpt1a* led to Impaired Wound Healing, Reduced Axonal Sparing and Worsened Locomotor Recovery in Mice After SCI, Effects Rescued by Exogenous β‐OHB

To further investigate the biological function of CPT1A in astrocytes following SCI, we generated astrocyte‐specific *Cpt1a* conditional knockout (CKO) mice (Figure S6A). Notably, histopathological evaluation at 14 dpi revealed that *Cpt1a* CKO in astrocytes led to lesion expansion, significant infiltration of CD68^+^ macrophages/microglia, and reduced neuronal survival (Figure 4A,B). Simultaneously, the proliferation of astrocytes (GFAP^+^SOX9^+^) at the perilesional site was decreased, and glial boundary formation was impaired (Figure 4C). Furthermore, *Cpt1a* CKO weakened the lipid metabolic capacity of astrocytes, resulting in lipid accumulation in astrocytes around the perilesional area (Figure 4D). However, supplementation with exogenous β‐OHB reversed these detrimental effects (Figure 4A–D). Subsequent functional assessments via hindlimb kinematic analysis showed that, compared to the control group, CKO mice exhibited reduced ankle motion, shortened stride length, decreased maximum iliac crest height, and attenuated hindlimb joint angle oscillations (Figure 4E,4F). Given that serotonergic neuromodulation in the lumbar circuits is crucial for locomotor recovery [36], we found that *Cpt1a* knockout significantly impaired the preservation of 5‐HT^+^ axons innervating lumbar motor neurons (Figure 4G). Electromyography (EMG) analysis further confirmed that *Cpt1a* CKO reduced the amplitude of motor‐evoked potentials (MEPs) in the gastrocnemius muscle (Figure 4H). Critically, administration of exogenous β‐OHB effectively abolished the functional and tissue impairment caused by the loss of astrocytic *Cpt1a* in mice post‐injury (Figure 4E–H). In primary astrocytes, β‐OHB supplementation reversed the reduction in astrocyte proliferation and migration caused by *Cpt1a* CKO (Figure S6B–D). As an alternative energy substrate, β‐OHB compensated for the requirement of CPT1A‐dependent ketogenesis, thereby restoring neuronal energy homeostasis, inhibiting oxidative stress, maintaining mitochondrial stability, and promoting neuronal survival (Figure S6E–J). In summary, astrocyte‐specific deletion of *Cpt1a* led to impaired tissue healing, axonal sparing, and functional recovery in mice after SCI, while exogenous β‐OHB supplementation effectively alleviated these impairments.

**FIGURE 4:**
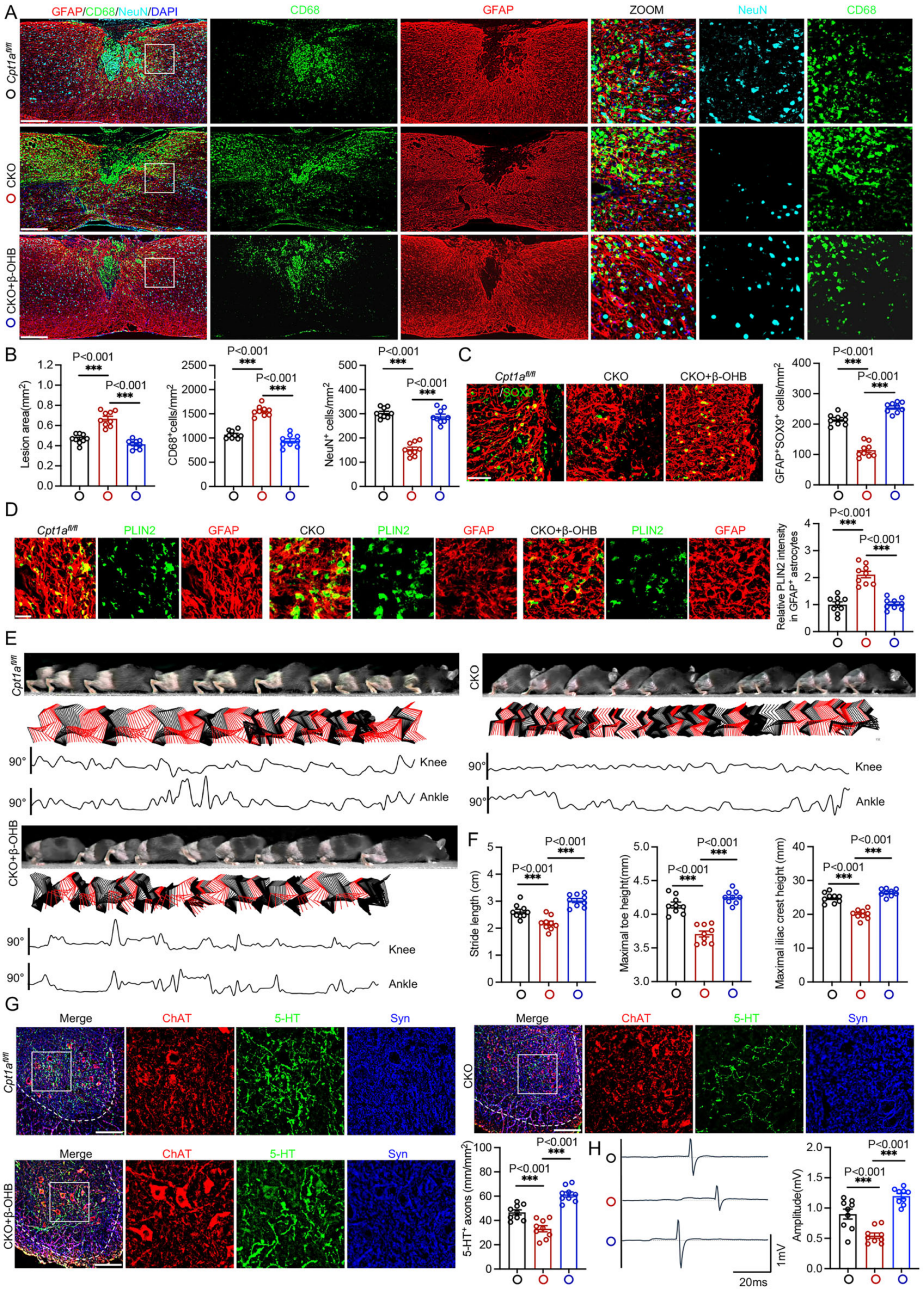
Astrocyte‐specific deletion of *Cpt1a* led to impaired wound healing, reduced axonal sparing and worsened locomotor recovery in mice after SCI, effects rescued by exogenous β‐OHB. (A,B) Representative images of CD68, GFAP, and NeuN co‐staining in the mouse spinal cord at 14 dpi for the specified groups, along with quantification of lesion area, CD68^+^ macrophages/microglia, and neurons (*n* = 9 per group). Scale bar = 500 µm. (C) Double staining of GFAP and SOX9 in the spinal cord of mice from specified groups, and quantification of GFAP^+^SOX9^+^ cells (*n* = 9 per group). Scale bar = 50 µm. (D) Double staining of GFAP and PLIN2 at 14 dpi, and quantification of PLIN2 fluorescence intensity in GFAP^+^ cells (*n* = 9 per group). Scale bar = 20 µm. (E) Representative gait images at 14 dpi, corresponding color‐coded hindlimb motion bar plots, and representative trajectories of hindlimb knee and ankle angle oscillations. (F) Quantification of stride length, maximal toe height, and maximal iliac crest height at 14 dpi (*n* = 9 per group). (G) Representative images of 5‐HT^+^ axons around ChAT^+^ motor neurons co‐stained with Syn in the lumbar ventral horn at 14 dpi (*n* = 9 per group). Scale bar = 50 µm. (H) Representative EMG recordings of the gastrocnemius muscle and quantification of the peak‐to‐peak amplitude of MEPs at 14 dpi (*n* = 9 per group). Data were analyzed using a one‐way ANOVA followed by post hoc Bonferroni correction (B–D, F–H).

### 2.5 Overexpression of CPT1A in Astrocytes Enhanced Tissue Repair after SCI

To further investigate the role of astrocytic CPT1A in vivo, we injected AAV‐CPT1A or the corresponding control AAV‐Con into SCI mice to induce astrocyte‐specific overexpression of CPT1A (Figure S7A). The results showed that ectopic overexpression of CPT1A promoted the proliferation and activation of astrocytes post‐injury (Figure S7B). Subsequent investigations revealed that, compared to the control group, CPT1A overexpression reduced the lesion area, inflammatory cell infiltration, and neuronal loss (Figure S7C,D), while also promoting astrocytic proliferation and glial boundary formation within the injury site (Figure S7E). Notably, in CPT1A‐overexpressing mice, the lipid metabolic capacity of astrocytes surrounding the lesion was enhanced, accompanied by a reduction in lipid accumulation (Figure S7F).

### 2.6 Astrocytic Ketogenesis Promotes Wound Repair and Lipid Metabolic Reprogramming, Forming a Positive Feedback Loop via H3K9bhb‐Dependent Epigenetic remodeling

β‐OHB, a key metabolic intermediate, has also been demonstrated to mediate histone lysine Kbhb modification, thereby linking metabolic reprogramming to epigenetic remodeling [29, 37]. Evaluation of histone Kbhb changes in astrocytes revealed a significant elevation of H3K9bhb levels post‐SCI (Figure 5A,B). We sorted spinal cord astrocytes from sham and SCI mice and performed genome‐wide ATAC‐seq and CUT&Tag analyses to identify target genes actively regulated by H3K9bhb in astrocytes after SCI. DeepTools analysis revealed that chromatin accessibility in astrocytes from SCI mice was significantly upregulated compared to the sham group (Figure 5C). We observed increased accessibility of proliferation‐ and migration‐related motifs (SOX9, STAT3, SOX2) and lipid metabolism‐related motif (TFE3) in injured astrocytes (Figure 5D). Furthermore, H3K9bhb binding peaks were significantly upregulated in SCI astrocytes (Figure 5E). GO analysis of the different H3K9bhb peaks at the promoter regions of target genes revealed significant enrichment in wound healing, glial cell proliferation/migration, lipid metabolism, and the TCA cycle in astrocytes after SCI (Figure 5F). Specifically, the genomic loci of key lipid metabolism‐related genes (*Acat2*, *Acadl*, *Acaa1a*, *Hmgcl*), proliferation and wound healing‐related genes (*Mki67*, *Pcna*, *Myc*, *Tgfb3*) exhibited higher chromatin accessibility and H3K9bhb peaks in astrocytes after SCI (Figure 5G). Further ChIP‐qPCR analysis confirmed that H3K9bhb levels at the promoters of these genes were significantly higher in injured astrocytes (Figure 5H). RT‐qPCR analysis further verified that the expression of these genes was significantly increased in astrocytes after SCI (Figure 5I). In *Cpt1a* CKO mice, astrocytes exhibited reduced H3K9bhb levels and decreased mRNA expression of above genes; however, supplementation with exogenous β‐OHB reversed these effects (Figure 5J–L). In *Cpt1a* CKO primary astrocytes, we co‐administered β‐OHB with the P300 inhibitor C646 and assessed the rescue effect by EdU proliferation and scratch wound healing assays. The results showed that exogenous β‐OHB could not rescue *Cpt1a* CKO phenotypes when H3K9bhb formation was blocked (Figure S8A,B). CPT1A overexpression in astrocytes led to significantly enhanced ketogenic flux and increased H3K9bhb occupancy at the promoters of lipid metabolism genes (Figure S8C–H). Critically, time‐course analyses (Figure S8E–H) not only support the causal direction from CPT1A to H3K9bhb‐mediated transcription but also substantiate the proposed feed‐forward loop. We further treated astrocytes with the ketogenesis inhibitor Hym and the Kbhb inhibitor C646. The results showed that the levels of global Kbhb and H3K9bhb were significantly reduced, and the mRNA levels of *Acat2*, *Acadl*, *Acaa1a*, *Hmgcl*, *Mki67*, *Pcna*, *Myc*, and *Tgfb3* were significantly downregulated (Figure S8I–N). In summary, these findings confirm that post‐SCI ketogenesis in astrocytes establishes a positive feedback loop through H3K9bhb‐dependent epigenetic remodeling, thereby promoting wound repair and lipid metabolic reprogramming.

**FIGURE 5:**
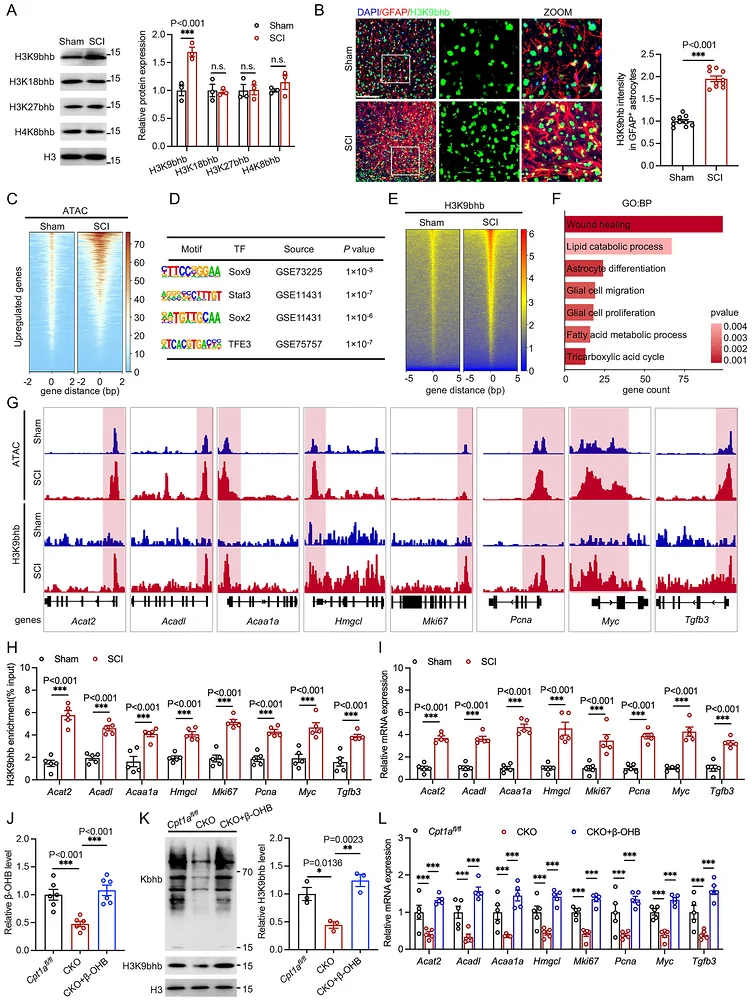
Astrocytic ketogenesis promotes wound repair and lipid metabolic reprogramming, forming a positive feedback loop via H3K9bhb‐dependent epigenetic remodeling. (A) Western blot analysis of histone Kbhb modifications in mouse spinal cord tissues (*n* = 3 per group). (B) Immunofluorescence analysis of H3K9bhb expression levels in spinal cord astrocytes of sham and SCI mice at 14 dpi (*n* = 10 per group). Scale bar = 100 µm. (C) DeepTools visualization of ATAC‐seq chromatin accessibility regions. (D) Motif analysis of genes with upregulated ATAC‐seq chromatin accessibility. (E) DeepTools visualization of H3K9bhb binding density. (F) GO enrichment analysis of DEGs characterized by both increased chromatin accessibility and enhanced H3K9bhb enrichment. (G) Genome browser analysis of ATAC‐seq and CUT&Tag signals for *Acat2*, *Acadl*, *Acaa1a*, *Hmgcl*, *Mki67*, *Pcna*, *Myc*, and *Tgfb3*. The red rectangle highlights the different peak region of the relevant genes. (H) ChIP‐qPCR analysis of the promoters of the indicated genes using anti‐H3K9bhb antibodies from sham and SCI mice at 14 dpi (*n* = 5 per group). (I) mRNA levels of specified genes from sham and SCI mice at 14 dpi (*n* = 5 per group). (J) Quantitative analysis of β‐OHB levels in astrocytes of the indicated groups (*n* = 6 per group). (K) Western blot analysis of Kbhb and H3K9bhb levels in astrocytes of the indicated groups (*n* = 5 per group). (L) RT‐qPCR analysis of mRNA levels of relevant genes in astrocytes of the indicated groups (*n* = 5 per group). Data were analyzed using a two‐tailed unpaired Student's *t*‐test (A, B, H, I) and one‐way ANOVA followed by post hoc Bonferroni correction (J‐L).

### 2.7 The Ketogenic Diet Promotes Tissue Repair after CNS Injury

Based on the critical role of β‐OHB in SCI repair, we further investigated the effect of exogenous β‐OHB administration on SCI recovery. The results showed that exogenous β‐OHB reduced the lesion area, inflammatory cell infiltration, and neuronal loss (Figure S9A,B), while promoting astrocyte proliferation and glial boundary formation at the injury site (Figure S9C). Concurrently, in mice receiving exogenous β‐OHB, the lipid metabolic capacity of perilesional astrocytes was enhanced, accompanied by reduced lipid accumulation (Figure S9D). Furthermore, to validate the protein stabilization and epigenetic effects of β‐OHB under physiological conditions, we treated primary astrocytes with β‐OHB at a physiologically attainable concentration (0.5 mM). The results demonstrated that β‐OHB at physiologically relevant concentrations was still capable of enhancing CPT1A stability and promoting epigenetic effects (Figure S9E,F). Ketogenic diet (KD), as an oral nutritional intervention, can significantly elevate systemic β‐OHB levels and offers advantages in long‐term adherence and clinical feasibility. We therefore further investigated the role of KD in CNS injury repair. Following the establishment of SCI, TBI, or MACO models, mice were subjected to a 2‐week KD intervention (Figure S10A). As expected, the KD group exhibited significantly higher blood ketone levels than the normal diet (ND) in SCI mice (Figure S10B). Moreover, KD substantially elevated overall β‐OHB levels and enhanced Kbhb modification in the injured tissues, accompanied by increased protein expression of CPT1A, SOX9, and GFAP in astrocytes (Figure S10C,D). KD‐treated SCI mice showed reduced lesion areas, decreased CD68^+^ macrophage/microglia infiltration, and a significant increase in surviving neurons at 14 dpi (Figure 6A,B). Compared to ND controls, KD enhanced astrocyte proliferation and boundary formation in the lesioned areas while reducing lipid accumulation in astrocytes surrounding the lesion (Figure 6C,D). Furthermore, to extend our findings and assess the broader therapeutic potential of KD, we leveraged two additional CNS injury models: TBI and stroke. In the TBI model, KD significantly decreased the lesion volume, reduced lipid accumulation in perilesional astrocytes, and promoted neuronal survival (Figure 6E–H). Similarly, in the MCAO model, KD reduced the infarct volume, attenuated lipid accumulation in astrocytes at the ischemic boundary, and protected surviving neurons in the ischemic zone (Figure 6I–L). In summary, KD effectively promotes tissue repair across multiple CNS injury models.

**FIGURE 6:**
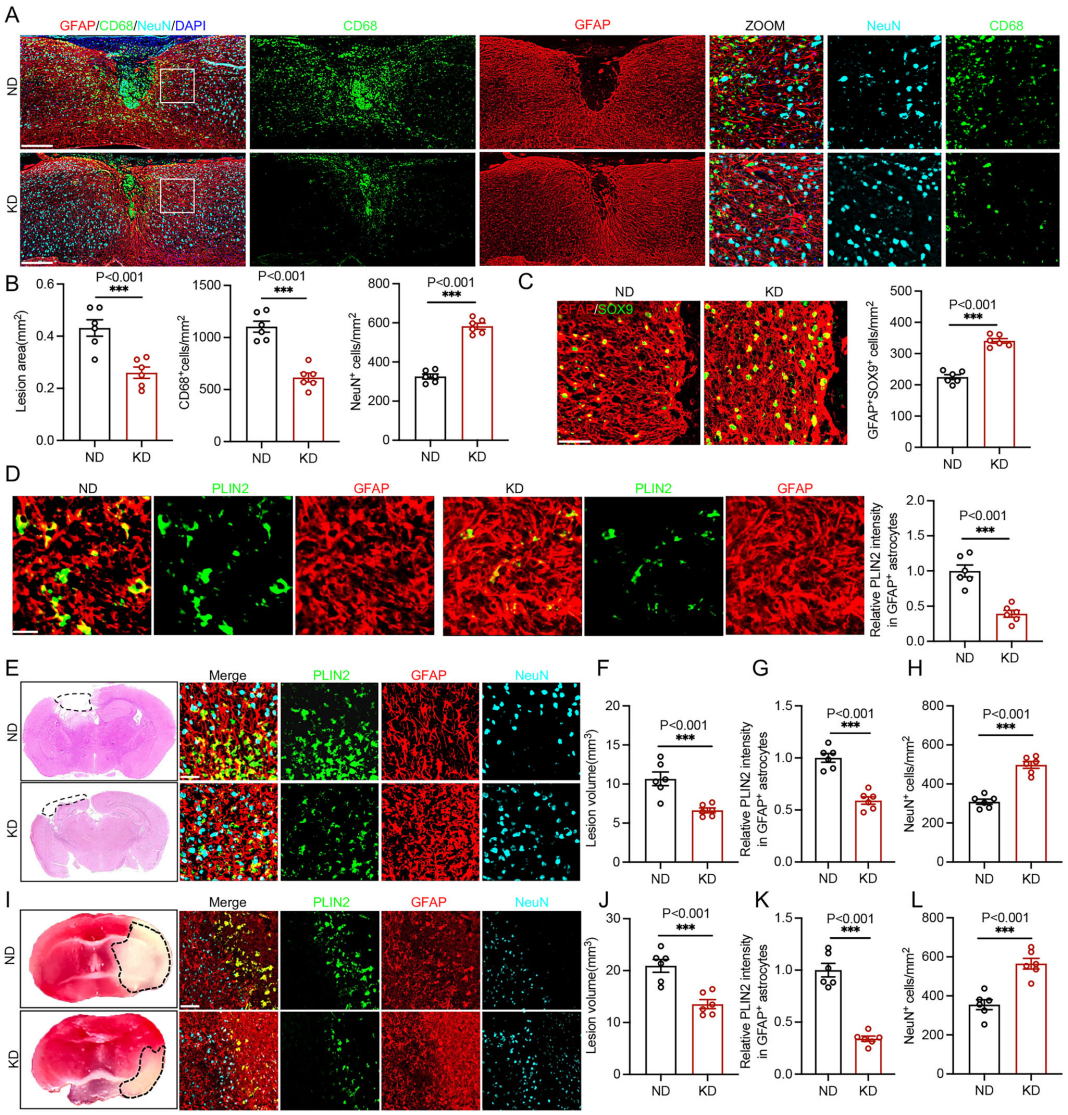
The ketogenic diet promotes tissue repair after CNS injury. (A,B) Representative images and quantification of CD68, GFAP, and NeuN co‐staining in the mouse spinal cord at 14 dpi of the ND and KD groups, along with quantification of lesion area, CD68^+^ macrophages/microglia, and neurons (*n* = 6 per group). Scale bar = 500 µm. (C) Double staining of GFAP and SOX9 in the spinal cord of ND and KD mice, and quantification of GFAP^+^SOX9^+^ cells (*n* = 6 per group). Scale bar = 50 µm. (D) Double staining of GFAP and PLIN2 in the spinal cord of ND and KD mice, and quantification of PLIN2 fluorescence intensity in GFAP^+^ cells (*n* = 6 per group). Scale bar = 20 µm. (E) Representative HE and immunofluorescence staining of GFAP/PLIN2/NeuN in the brain tissues of TBI mice at 14 dpi of the ND and KD groups. Scale bar = 20 µm. (F–H) Quantification of brain lesion area (F), PLIN2 fluorescence intensity in GFAP^+^ cells (G), and surviving neurons (H) of the ND and KD mice at 14 dpi after TBI (*n* = 6 per group). (I) Infarct volume and immunofluorescence staining of GFAP/PLIN2/NeuN in the brains of the ND and KD mice at 14 dpi after MCAO. Scale bar = 50 µm. (J–L) Quantification of brain lesion area (J), PLIN2 fluorescence intensity in GFAP^+^ cells (K), and surviving neurons (L) of the ND and KD mice at 14 dpi after MCAO (*n* = 6 per group). Data were analyzed using a two‐tailed unpaired Student's *t*‐test (B–D, F–H, J–L).

### 2.8 High Levels of β‐OHB in the CSF of SCI Patients Indicate better Tissue Preservation and Functional Recovery

To further explore the potential role of β‐OHB in clinical SCI patients, we collected cerebrospinal fluid (CSF) samples and imaging data from patients admitted with acute SCI and conducted a one‐year clinical follow‐up to assess functional recovery (Figure 7A and Table S1). The results showed that β‐OHB levels in the CSF of SCI patients were significantly higher than those in the control group (Figure 7B). Patients with complete motor paralysis (American Spinal Injury Association [ASIA] grade A/B) who had higher CSF β‐OHB levels were more likely to achieve improved functional recovery after one year of follow‐up (Figure 7C). Subsequent integration with radiological analysis revealed that CSF β‐OHB content was significantly negatively correlated with the area of hyperintensity lesions (reflecting edema or compression) on MRI T2‐weighted imaging (Figure 7D). Moreover, in the analysis of fractional anisotropy (FA) values from diffusion tensor imaging (DTI), a key indicator of axonal integrity, β‐OHB levels showed a significant positive correlation with FA values (Figure 7E,F). Since high CSF levels of GFAP and NF‐L typically predict poor functional prognosis in SCI patients [38, 39], we found that CSF β‐OHB content was also significantly negatively correlated with GFAP and NF‐L levels (Figure 7G,H). To remove the potential confounding effects of ASIA grade, neurological level of injury, age, sex, and time to sampling on the correlation analyses, we performed multivariable linear regression to further examine the associations between CSF β‐OHB and four continuous outcome measures (T2 hyperintensity area, FA, GFAP, NF‐L), with baseline ASIA grade (A/B/C/D), neurological level (cervical/thoracic), age, sex, and injury‐to‐sampling interval included as covariates. β‐OHB remained a significant predictor in all models (all *p* < 0.001). These results indicate that the associations between CSF β‐OHB and outcome measures remained robust after adjusting for the above covariates (Figure S11A–D). In summary, these clinical findings collectively demonstrate the neuroprotective role of β‐OHB, suggesting that high CSF β‐OHB levels serve as a critical biological indicator of favorable neurological prognosis in SCI patients.

**FIGURE 7:**
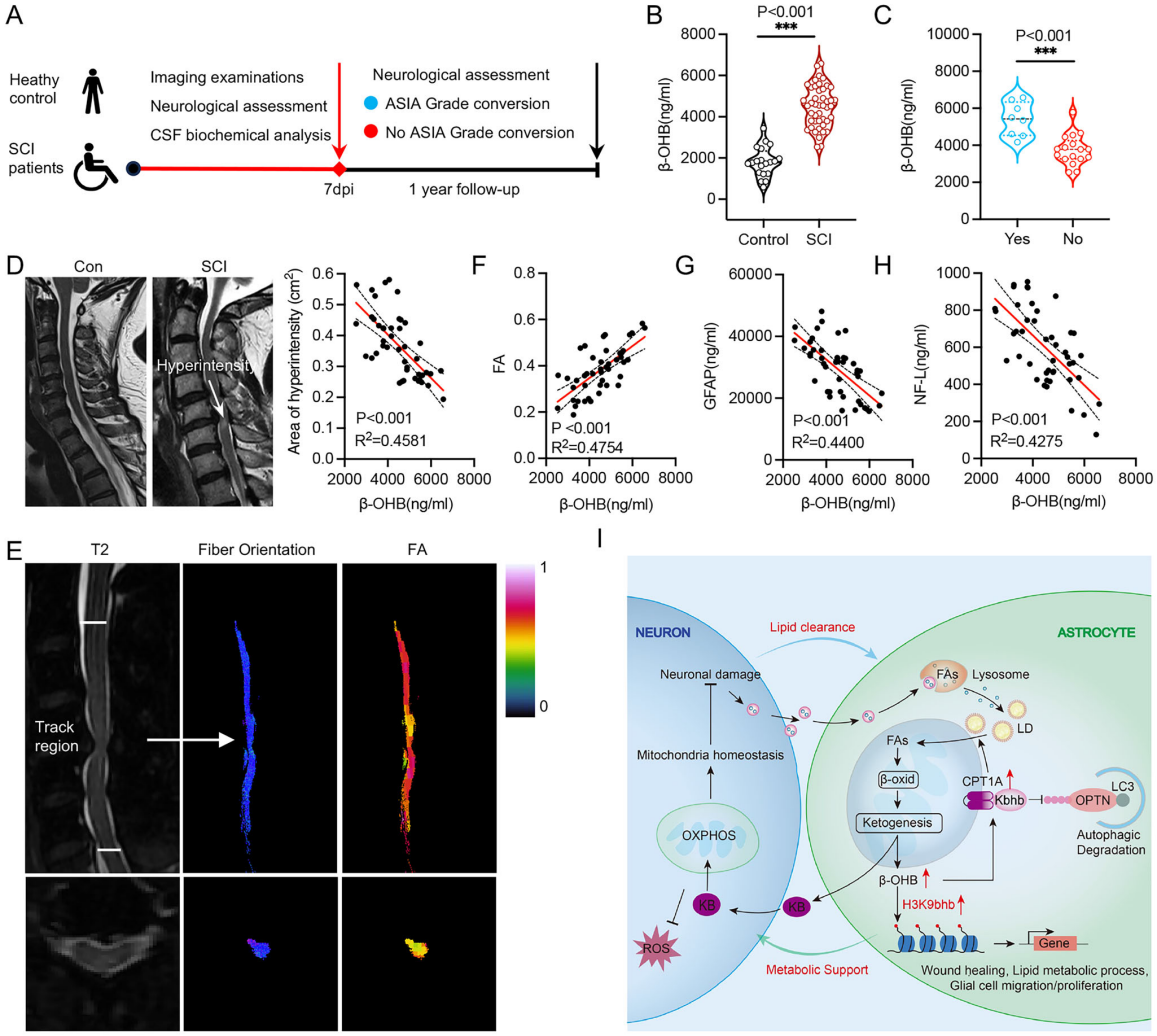
High levels of β‐OHB in the CSF of SCI patients indicate better tissue preservation and functional recovery. (A) Schematic illustrating the clinical study design for control and SCI patients. (B) Measurement of β‐OHB content in CSF (healthy controls, *n* = 20; SCI patients, *n* = 45). (C) Analysis of CSF β‐OHB levels based on motor recovery in ASIA grade A/B SCI patients (ASIA grade improvement, *n* = 8; No ASIA grade improvement, *n* = 17). (D) Representative MRI T2‐weighted images from healthy controls and SCI patients, and correlation analysis between hyperintensity areas in T2‐weighted MRI and β‐OHB levels in SCI patients (*n* = 45). (E) Representative DTI images of SCI patients. (F) Correlation analysis between FA values and β‐OHB levels in SCI patients (*n* = 45). (G,H) Correlation analysis between CSF GFAP (G)/NF‐L (H) levels and β‐OHB levels in SCI patients (*n* = 45). (I) Schematic presentation of the study. Data were analyzed using a two‐tailed unpaired Student's *t*‐test (B, C) and Pearson correlation analysis (D, F–H).

## 3 Discussion

Recent studies employing scRNA‐seq and multi‐omics approaches have begun to characterize molecular expression profiles at the astrocyte border; however, the metabolic and epigenetic regulatory mechanisms operating within these specialized regions remain poorly understood. This study demonstrates that reactive astrocytes at the lesion border following SCI exhibit enhanced lipid metabolism and exert neuroprotective effects through coordinated metabolic and epigenetic remodeling (Figure 7I). From a translational perspective, we show that KD promotes wound repair after CNS injury, further supporting its potential as a promising therapeutic strategy in neurological disorders.

For decades, astrocyte reactivity was considered a uniform, functionally insignificant reaction to tissue damage, primarily serving as a mere marker of pathology. This perception has been radically revised by recent studies demonstrating that modulating astrocyte reactivity can significantly influence disease outcomes [7, 40]. Once thought to be homogeneous and passive, reactive astrocytes are now recognized as a heterogeneous, context‐dependent, and powerful determinant of pathology. They can modulate CNS inflammation, preserve tissue integrity, contribute to circuit reorganization and axon regeneration, and in certain contexts, exert harmful effects through the loss of normal functions or the gain of detrimental properties [41, 42, 43]. The role of reactive astrocytes and the ensuing glial scar in recovery is complex. The conventional wisdom holds that the glial scar acts as a physical barrier and secretes chondroitin sulfate proteoglycans, which inhibit axonal growth and regeneration [10, 44]. Furthermore, astrocytes acquire pro‐inflammatory transcriptional profiles after neurological injury and disease [45, 46, 47]. Prior research has confirmed that the excessive inflammatory response mediated by reactive astrocytes directly exacerbates CNS damage [48, 49, 50, 51]. By secreting chemokines that recruit peripheral immune cells, pro‐inflammatory astrocytes can further aggravate tissue injury [52, 53]. This body of work underscores the detrimental potential of reactive astrocytes following CNS disorders. Challenging the traditional view, a substantial and growing body of evidence indicates that preventing or attenuating astrocyte border formation disrupts wound healing, leads to widespread inflammation, impairs blood‐brain barrier restoration, exacerbates neural tissue loss and hampers functional recovery [13, 54, 55, 56]. The newly generated and reprogrammed astrocytes around lesions perform an essential parenchymal function by surrounding and isolating the stromal scar tissue, thereby reestablishing CNS tissue integrity. These findings argue that astrocyte borders should no longer be simplistically labeled as scars. The appealing concept of achieving scar‐free wound healing in the adult CNS is supported by observations in mammalian neonates, where microglia and astrocytes undergo high levels of proliferation, enabling a rapid glia‐led repair process that avoids fibrosis and effectively promotes neural circuit regeneration [57]. These wound‐repair astrocytes are the primary source of neuroprotective borders that restore tissue integrity around CNS lesions by separating neural parenchyma from stromal and immune cells, mirroring the organization in the healthy CNS. Our previous work has also demonstrated that promoting specific reactive astrocyte responses is beneficial for wound repair and functional recovery after SCI [16, 58, 59]. Therefore, a nuanced understanding of the regulatory mechanisms governing astrocyte reactivity in different contexts is crucial for identifying strategies to selectively inhibit detrimental responses while promoting beneficial ones.

Metabolic pathways are increasingly recognized as key modulators of astrocyte reactivity across multiple neurological conditions, making them attractive targets for therapeutic intervention [14, 60]. As the two most abundant and energetically pivotal cell types in the brain, neurons and astrocytes are metabolically coupled not only in glucose oxidation but also in lipid metabolism [15, 61]. Astrocytes support neuronal function through various mechanisms, including fuel supply, cholesterol provision, release of neurotrophic factors, and mitochondrial transfer [20, 62]. Although the brain primarily relies on glucose as its fuel, lipids are structurally and functionally indispensable. While fatty acids (FAs) serve as essential building blocks for membranes and substrates for energy storage, elevated FA levels are associated with cellular toxicity and metabolic dysfunction. Tight control of FA concentrations is especially critical in the brain due to its high energy demand and limited capacity for FA catabolism. Neurons have minimal ability to break down FAs, and excess FAs can induce mitochondrial uncoupling and dysfunction. Therefore, precise regulation and timely clearance of neuronal free FAs are vital for protecting against neurodegeneration [21, 26]. In peripheral tissues, two complementary pathways including FAO and sequestration of FAs into intracellular LDs serve to alleviate intracellular FA burden and prevent lipotoxicity. Disruption of FAO or OXPHOS has been linked to neuroinflammation, neurodegeneration and cognitive decline [63, 64]. In the brain, LDs accumulate in glial cells under conditions of neuronal oxidative stress and during aging [65, 66], a phenomenon our findings extend to the context of CNS injury. Growing evidence indicates that astrocytes can synthesize KBs owing to their capacity to oxidize FAs, suggesting the existence of an intrinsic brain system for local KB production and delivery [67, 68]. The metabolite composition of CSF directly reflects the metabolic homeostasis of the CNS microenvironment [69]. Ketone bodies synthesized by astrocytes are released into the extracellular space via monocarboxylate transporters, supplying energy to neurons and potentially acting as direct antioxidants to reduce mitochondrial reactive oxygen species production [70, 71, 72]. In mammals, the brain appears to depend on ketone body metabolism during two key life stages: postnatal development and aging, and cerebral microdialysis experiments have confirmed that brain ketogenesis can be activated in situ [73, 74]. Therefore, the compensatory elevated CSF β‐OHB concentrations observed after SCI largely reflect this compensatory upregulation of astrocytic ketogenic activity in response to stress. The model of metabolic coupling between astrocytes and neurons after CNS injury proposed in this study expands the relevance of ketone body metabolism in neuroscience.

KBs, comprising β‐OHB, acetoacetate and acetone, feature β‐OHB as the most abundant form in this group. Historically, KBs, primarily synthesized in hepatic mitochondria and significantly elevated during prolonged fasting, KD or certain pathological states, have been widely recognized as energy substrates [75, 76]. Numerous studies indicate that β‐OHB supplementation can prevent or ameliorate symptoms in neurodegenerative disorders, cardiovascular diseases and cancers, among other conditions [77, 78, 79]. Recently, KB metabolism has attracted growing interest, with emerging evidence showing that KBs function not only as alternative energy sources but also as metabolic signaling molecules. Specifically, KBs act as chemical donors for Kbhb, a post‐translational modification targeting both histone and non‐histone proteins, thereby influencing chromatin architecture, gene transcription and cellular signaling pathways [80, 81, 82]. Nevertheless, the signaling roles of KBs in the context of CNS injury remain poorly understood. Metabolic adaptations are often coupled with epigenetic reprogramming to orchestrate transcriptional responses. The contribution of epigenetics to astrocyte‐driven pathology in neurological diseases has recently gained renewed attention [83, 84, 85, 86]. For instance, in astrocytes exposed to pro‐inflammatory cytokines, metabolic remodeling leads to acetyl‐CoA production, which facilitates histone acetylation and chromatin relaxation, thereby amplifying NF‐κB‐driven pro‐inflammatory gene expression [24]. This illustrates how inflammation‐induced metabolic shifts in astrocytes can reinforce their pro‐inflammatory phenotype. Another study revealed that epigenetic alterations in astrocytes following TBI promote neurodegeneration by exacerbating neuroinflammation and dysregulating immune responses [52]. In this study, we demonstrate that β‐OHB‐mediated Kbhb modification inhibits the autophagic degradation of CPT1A and remodels the epigenetic landscape of astrocytes through H3K9bhb, thereby establishing a lipid metabolic reprogramming loop that sustains the activation of reactive astrocytes after SCI. Although H3K9bhb driven by β‐OHB is a global transcriptional activation mark, previous studies have demonstrated that its genomic deposition is not indiscriminate [74]. Through precise recognition by specific epigenetic “reader” proteins and spatial cooperation with key transcription factors at defined promoter or enhancer regions [87, 88, 89], H3K9bhb is capable of establishing a highly specific targeting network.

The KD, a high‐fat, moderate‐protein and low‐carbohydrate nutritional intervention, is considered a safe and feasible therapeutic strategy for multiple disorders [90, 91, 92]. Our findings further support the utility of KD in reducing lipid‐laden reactive astrocytes and promoting tissue repair following CNS injury. However, given that KD exerts neuroprotection through diverse mechanisms [93, 94], the specific pathways underlying its beneficial effects in CNS injury warrant further elucidation.

This study has several limitations that should be acknowledged. First, the heterogeneity of astrocytes residing at the lesion border has not been fully characterized. Second, the potential contributions of other metabolic pathways in reactive astrocytes to the tissue repair process remain largely unexplored. Third, the mechanisms by which astrocytes coordinate signaling with other cell types to facilitate wound healing are still not well understood.

## 4 Materials and Methods

### 4.1 Human Tissue Samples

This study enrolled patients with acute traumatic cervical SCI who had no concurrent injuries. Within 7 dpi, we collected CSF samples, conducted neurological assessments, and performed imaging examinations (MRI and DTI). Subsequently, all SCI patients received standardized surgical intervention and postoperative rehabilitation from a single clinical team. The control group consisted of patients undergoing intrathecal anesthesia who were free of neurological diseases. CSF samples were obtained from all subjects as we previously described [36]. The ethics committee at the First Affiliated Hospital of Nanjing Medical University approved this study (2024‐SR‐1109), and written informed consent was obtained from all participants prior to tissue collection. Detailed demographic and clinical information for both SCI patients and controls are provided in Table S1.

### 4.2 Mice

Adult male or female WT C57BL/6 mice (8‐12 weeks old) were obtained from the Animal Center of Nanjing Medical University. Astrocyte‐specific *Cpt1a* knockout mice were generated by crossing *Cpt1a^fl/fl^* mice (S‐CKO‐18358, Cyagen Biosciences) with *Aldh1l1^CreERT2^* mice (C001288, Cyagen Biosciences). Eight‐week‐old mice were intraperitoneally injected with tamoxifen (HY‐13757A, MCE) at a dose of 75 mg kg^−1^ body weight once daily for 5 consecutive days [52]. One week after treatment, tamoxifen‐inducible, *Aldh1l1*‐driven astrocyte‐specific *Cpt1a* conditional knockout mice were generated. Littermate *Cpt1a^fl/fl^* mice treated with tamoxifen served as the control group. All mice were housed in a temperature‐controlled facility (23°C, 60% humidity) with a 12 h light/dark cycle and ad libitum access to food and water. Throughout the experimental period, manual bladder expression was performed twice daily alongside standard animal care. All experimental protocols were approved by the Institutional Animal Care and Use Committee of the First Affiliated Hospital of Nanjing Medical University (IACUC‐2401012) and were conducted in accordance with the Guide for the Care and Use of Laboratory Animals.

For continuous delivery of β‐OHB (HY‐113378; MCE), ALZET osmotic pumps (Model 2002; DURECT Corp., Cupertino, CA, USA) were implanted subcutaneously during surgery. β‐OHB (1.6 mmol/kg/d in saline) or vehicle (saline) was infused at a constant rate of 0.5 µl/h for 14 days [95, 96].

### 4.3 Bioinformatics Analysis

#### 4.3.1 Data Download and Processing

To elucidate the metabolic landscape and transcriptional dynamics in astrocytes following SCI, we reanalyzed publicly available mouse spinal cord snRNA‐seq/scRNA‐seq and ST datasets (GSE234774; GSE205029). Batch effect correction was performed using Harmony, and the integrated Harmony embeddings and subsequent analyses followed the standard Seurat R workflow. High‐dimensional data were then projected into two‐dimensional space for visualization using UMAP. Cluster‐specific marker genes were identified using the FindAllMarkers function (Wilcoxon rank‐sum test), and cell‐type identities were assigned by integrating known lineage‐specific markers with SingleR‐mediated automated annotation. To integrate the scRNA‐seq atlas with the ST data, we utilized Robust Cell Type Decomposition (RCTD) to deconvolve spatial barcodes into mixtures of one or more cell types. Smooth patterns of cell‐type abundance were reconstructed through two‐dimensional local weighted regression of the deconvolution weights. Additionally, discrete cell‐type assignments were attributed to each spatial barcode based on the maximum deconvolution weight derived from the RCTD model.

### 4.4 Metabolic Analysis of Astrocytes

To assess the metabolic adaptability of astrocytes within the scRNA‐seq dataset, the R package scMetabolism was utilized to quantify metabolic activity in both sham and injured spinal cords. Subsequently, GSEA method was employed to estimate activity scores for KEGG pathways and comprehensive genomic metabolic gene sets, thereby characterizing injury‐induced metabolic shifts in the astrocytic population. For the ST data, gene set enrichment scores were calculated using the AddModuleScore function in Seurat, enabling the high‐resolution spatial mapping of metabolic signatures within the microenvironment of the injured spinal cord.

### 4.5 GSEA

To evaluate the changes in metabolic pathways after injury, differentially expressed genes (DEGs) in astrocytes at multiple time points after SCI were first screened and compared with the sham group. A ranked list was then generated based on the fold change and *p*‐value of all detected DEGs. Subsequently, GSEA analysis was performed using the clusterProfiler package to identify pathways significantly enriched in the Hallmark gene sets obtained from MSigDB.

### 4.6 Surgical Procedures

#### 4.6.1 SCI

SCI was performed at the thoracic level (T10) similar as previous described [97, 98]. Briefly, mice were anesthetized via isoflurane inhalation, followed by a T9‐T11 laminectomy to fully expose the spinal cord. A bilateral dorsal crush injury was induced at the T10 level using 0.3‐mm forceps applied for 10 s at a depth of approximately 1.1 mm over the central canal. This lesion preserves only a portion of the ventral white matter, resulting in severe and permanent neurological deficits. Postoperatively, mice were maintained on a warming pad until full recovery from anesthesia. Penicillin was administered intramuscularly for three days (twice daily), and manual bladder expression was performed twice daily until the restoration of spontaneous voiding. For the sham‐operated control group, only the T10 laminectomy was performed.

#### 4.6.2 TBI

The TBI mouse model was established using a controlled cortical impact device (68097, RWD, China). Anesthetized mice were secured in a stereotaxic frame, and the skin was incised following disinfection. A 4‐mm diameter craniotomy was performed over the right parietal cortex (between bregma and lambda, 1 mm lateral to the midline) using a portable drill. With the dura mater kept intact, a cortical contusion was induced using a 2‐mm diameter round‐tipped steel impactor at a velocity of 1.5 m s^−1^, a depth of 1.5 mm, and a dwell time of 0.5 ms. After the procedure, the bone flap was sealed with bone wax and the scalp was sutured. The sham group underwent the same craniotomy procedures without receiving the cortical impact. All animals were monitored in a 37°C recovery cage post‐surgery until they were fully awake and ambulatory.

#### 4.6.3 MCAO

Anesthetized mice were secured in a stereotaxic frame. The left common carotid artery (CCA) was exposed through a midline neck incision. The CCA and external carotid artery (ECA) were ligated, and a microvascular clip was used to temporarily block the retrograde flow of the internal carotid artery (ICA). A silicone‐coated 6‐0 nylon monofilament was then inserted through the CCA incision and advanced to the origin of the middle cerebral artery (MCA). Successful occlusion was confirmed by monitoring cerebral blood flow changes using a laser Doppler flowmeter. After 35 min of ischemia, the filament was slowly withdrawn to achieve reperfusion, and the wound was sutured. The sham group underwent the same surgical steps, except that the filament was withdrawn immediately after insertion without inducing ischemia.

### 4.7 Hindlimb Kinematic Analysis

For detailed hindlimb kinematic analysis, the hair on the hindlimbs of the mice was first removed to ensure clear visualization of anatomical landmarks. Ground‐walking capability was assessed by placing the mice in a transparent runway (80 cm long, 4 cm wide), with the external environment excluding strong light, noise, and low temperatures to avoid interference. For each animal, a high‐speed camera (120 fps) was used to record 6–10 consecutive gait cycles within the central 40‐cm region, while cycles involving head rotation were excluded. The recorded videos were analyzed offline using DeepLabCut to automatically track the coordinates of anatomical landmarks, including the iliac crest, hip, knee, ankle, and toe. Subsequently, kinematic parameters were quantitatively analyzed in a double‐blind manner, including maximum iliac crest and toe height, stride length, and joint angle oscillation (knee and ankle). To demonstrate locomotor performance, representative serial images capturing different phases of the gait cycle (stance and swing phases) were extracted and sequences were synthesized using Adobe Photoshop. Finally, stick‐point representations of hindlimb trajectories were generated using MATLAB to visualize motor coordination. Animals were randomly assigned to experimental groups using a random number table prior to SCI induction. Experimenters were blinded to group allocation during all behavioral assessments. Offline analysis of video data was performed independently by two investigators blinded to group allocation.

### 4.8 EMG

MEPs were assessed using an EMG test (DreamLab Technologies Shanghai Co., Ltd, China). Briefly, mice were anesthetized, and the spinal cord was surgically re‐exposed at the previous lesion site while body temperature was maintained via a heating pad. The stimulation electrodes were positioned at the rostral end of the exposed spinal cord, whereas the recording electrodes were inserted into the mid‐belly of the medial gastrocnemius. A grounding electrode was placed subcutaneously. A single electrical stimulus (0.5 mA, 0.5 ms duration, 1 Hz) was applied to evoke MEPs. The peak‐to‐peak amplitude of the MEPs was quantified to evaluate hindlimb nerve conduction and motor function.

### 4.9 KD Models

The mice were fed either a ND (carbohydrate/fat/protein = 80/10/10, XTKDCON) or a KD (carbohydrate/fat/protein = 0/90/10, XTKD01) for two weeks. The KD was stored at −20 °C and thawed to room temperature before feeding to prevent feeding inhibition or diarrhea caused by low‐temperature fodder. Blood β‐OHB levels were measured via tail vein blood sampling on days 0, 3, 7, and 14. After 2 weeks, the mice were sacrificed, and spinal cord and brain tissues were harvested, snap‐frozen in liquid nitrogen, and stored at −80°C for further analysis.

### 4.10 Administration of AAV In Vivo

The administration of AAV was performed as we previously described [58]. The plasmids pAAV‐GfaABC1D‐CPT1A‐3×FLAG‐WPRE (AAV‐CPT1A, Obio Technology) was used to specifically increase the exogenous expression of CPT1A in vivo. while the control vector pAAV‐GfaABC1D‐MCS‐WPRE (AAV‐Con) was used as a control. Following the establishment of the SCI model, mice were secured in a stereotaxic frame with a spinal cord adapter. Under an operating microscope, viral vectors (5 × 10^9^ vg; 1 µl) were injected into the four corners of the SCI site at a depth of 1 mm using a 10‐µl NeurosTM syringe (Hamilton Company, Reno, NV, USA) connected to a micro‐infusion pump controller at a rate of 200 nl min^−1^. After each injection, the needle was left in place for 3 min before being slowly withdrawn. Postoperatively, mice were placed on a 37°C warming pad until they fully recovered from anesthesia and received routine postoperative care.

### 4.11 Immunofluorescence Staining

Spinal cord or brain samples were fixed in 4% paraformaldehyde. Subsequently, coronal brain sections (30 µm) or sagittal spinal cord sections (15 µm) were permeabilized with 0.1% Triton X‐100 for 30 min at room temperature and blocked with 5% donkey serum for 2 h. The sections were then incubated overnight at 4°C in a humidified chamber with specific primary antibodies. The following day, the samples were washed three times with phosphate‐buffered saline containing 0.1% Tween‐20 for 5 min per wash. The samples were then incubated with fluorescence‐conjugated secondary antibodies for 1 h at room temperature in the dark. After additional washing, nuclei were counterstained with DAPI for 10 min followed by mounting. The primary and secondary antibodies included mouse/rabbit anti‐GFAP (GB12096, GB11096, Servicebio), rabbit anti‐CD68 (28058‐1‐AP, Proteintech), mouse anti‐CPT1A (66039‐1‐Ig, Proteintech), mouse anti‐NeuN (ab104224, Abcam), rabbit anti‐NeuN (GB11138, Servicebio), CoraLite 594‐conjugated GFAP antibody (CL594‐260190, Proteintech), mouse anti‐Perilipin‐2 (15294‐1‐AP, Proteintech), mouse anti‐SOX9 (67439‐1‐Ig, Proteintech), rabbit anti 5‐HT (20080, Immunostar), Mouse anti‐ChAT (66816‐1‐Ig, Proteintech), CoraLite 647‐conjugated rabbit polyclonal anti‐Syn (CL647‐17785, Proteintech), rabbit anti‐H3K9bhb (PTM‐1250RM, PTM Bio), Alexa Fluor488‐conjugated goat anti‐rabbit IgG (111‐545‐003, Jackson ImmunoResearch), Alexa Fluor594‐conjugated goat anti‐mouse IgG (115‐585‐003, Jackson ImmunoResearch), and Alexa Fluor647‐conjugated goat anti‐mouse IgG (115‐605‐003, Jackson ImmunoResearch). Brain and spinal cord sections were visualized using a THUNDER imaging system (Leica Microsystems GmbH, Wetzlar, Germany).

### 4.12 Measurement of β‐OHB

Blood samples were collected from the tail vein of treated mice. Plasma was separated by centrifugation at 1500 g for 15 min at 4°C and stored at −80°C until thawed for assay. Spinal cord tissues were homogenized and sonicated three times on ice using a high‐intensity ultrasonic processor. Debris was removed by centrifugation at 12 000 g for 10 min at 4°C, and the supernatants were collected for β‐OHB detection. The β‐OHB levels in plasma, and spinal cord were quantified using the Beta‐Hydroxybutyrate Assay Kit (ab83390, Abcam) following the manufacturer's instruction.

### 4.13 Measurement of GFAP and NF‐L in CSF

CSF levels of NF‐L and GFAP were measured using the Simoa HD‐X platform (Quanterix, MA, USA). Analytes were quantified using the Simoa NF‐light Advantage Kit (103186, Quanterix) and Simoa GFAP* Discovery Kit (102336, Quanterix), following the manufacturer's instructions. To ensure concentrations fell within the measurable range, CSF samples were diluted 1000‐fold (NF‐L) and 100 000‐fold (GFAP). The 100 000‐fold dilution was achieved through two serial 1:100 steps followed by a final 1:10 dilution using the provided dilution buffer.

### 4.14 Cell Culture

For primary astrocyte culture, brains from neonatal C57BL/6J mice were rapidly dissected, and the cerebral cortex was isolated and stripped of meninges before being cut into small pieces (0.5–1 mm^3^). The tissue was then digested with 0.25% trypsin‐EDTA (25200056, Thermo Fisher, USA) 37°C with shaking for 20 min. After neutralization with complete DMEM/F12, the cell suspension was filtered through 70‐µm strainers to remove debris. Following centrifugation, the cells were resuspended in DMEM and seeded into poly‐L‐lysine‐coated T75 flasks. Once the cells reached confluency, the astrocytes were shaken at 240 rpm for 6 h, rinsed twice with PBS, and then digested and seeded for follow‐up experiments.

For primary neuronal culture, the cerebral cortex was dissected and cut into small pieces, which were digested with papain (HY‐P1645, MCE) and DNase (9003‐98‐9, Aladdin), filtered, and seeded in poly‐D‐lysine‐coated dishes. The cells were cultured using the B‐27^TM^ Plus Neuron Culture System (A3653401, Thermo Fisher, USA) at 37°C in a 5% CO_2_/95% air atmosphere. The medium was replaced every 2 days.

### 4.15 MACS

To obtain a high‐quality single‐cell suspension from spinal cord tissue, cellular debris was first removed using a debris removal solution (130‐109‐398, Miltenyi Biotec). Subsequently, to eliminate contaminating erythrocytes, the cell pellet was resuspended in red blood cell lysis solution (130‐094‐183, Miltenyi Biotec) and incubated for 10 min at 4°C to ensure complete hemolysis. The reaction was quenched by adding a 10‐fold volume of PEB buffer (D‐PBS containing 0.5% BSA and 2 mM EDTA). The suspension was then centrifuged at 300 g for 10 min at 4°C, and the supernatant was completely aspirated. The final cell pellet was resuspended in an appropriate buffer for downstream magnetic cell separation. Following the manufacturer's protocol, astrocytes were labeled with anti‐ACSA‐2‐conjugated MicroBeads (130‐096‐054, Miltenyi Biotec) and isolated from the spinal cord single‐cell suspension using a MACS multistage separator.

### 4.16 Metabolic Flux Analysis

Primary astrocytes were cultured in a chemically defined tracer medium consisting of glucose‐free DMEM (11966025, Gibco) supplemented with [U‐^13^C_16_] palmitic acid (CLM‐409‐0.5, Cambridge Isotope Laboratories, Inc.) for 24 h at 37°C. Following the labeling period, metabolites were extracted and analyzed using an ultra‐high‐performance liquid chromatography‐high‐resolution mass spectrometry‐based metabolic flux platform.

### 4.17 Measurement of OCR

OCR in primary neurons and astrocytes was measured using a Seahorse XFe96 Extracellular Flux Analyzer (Agilent Technologies). Primary neurons or astrocytes were seeded at an appropriate density into Seahorse XF96 cell culture microplates and treated according to the experimental requirements. During the assay, 2 µM oligomycin, 1 µM FCCP and 1 µM rotenone/antimycin A were sequentially injected through the automated drug ports. OCR changes were recorded at each stage to evaluate basal respiration, ATP production, and maximal respiratory capacity.

To measure the FAO capacity of primary astrocytes, astrocytes isolated from the mouse spinal cord were incubated for 12 h in substrate‐limited medium (DMEM base medium supplemented with 0.5 mM glucose, 1.0 mM GlutaMAX, 0.5 mM carnitine, and 1% FBS, pH 7.4). On the day of the assay, the medium was replaced with FAO assay buffer, and the cells were incubated in a CO_2_‐free incubator at 37°C for 1 h. Prior to the start of the measurement, Palmitate‐BSA was added to each well. Subsequently, oligomycin, FCCP, Etomoxir (10 µM, added after OCR reached its peak), and rotenone/antimycin A were injected sequentially. The oxygen consumption contributed by FAO was evaluated by comparing the differences in OCR following Etomoxir treatment.

### 4.18 Cell Viability and Cell Death

The cell viability of primary neurons was evaluated using the CCK‐8 assay kit (CK04, Dojindo). Neuronal suspensions were seeded into 24‐well plates at a density of 50 000 cells well^−1^. Following the experimental treatments, 10 µL of CCK‐8 solution was added to each well, and the plates were incubated for an additional 2 h. Finally, the absorbance at 450 nm was measured using a microplate reader (ELx800, Bio‐Tek) for quantitative analysis.

For cell death detection, primary neurons were seeded in 24‐well plates and treated according to the experimental protocol. The culture medium was aspirated, and the cells were washed with PBS. Subsequently, 250 µL of Calcein‐AM/PI detection working solution (L3224, Thermo Fisher) was added to each well and incubated at 37°C for 30 min in the dark. Immediately after incubation, imaging was performed using the THUNDER Imaging System (Leica, Germany).

### 4.19 ROS

ROS levels in primary neurons were detected using a Reactive Oxygen Species Assay Kit (S0033S, Beyotime). First, primary neurons were seeded in 24‐well plates and treated according to the experimental requirements of different groups. During the assay, the culture medium was removed, and DCFH‐DA probe diluted in serum‐free medium (1:1000, 10 µM) was added and incubated at 37°C in the dark for 30 min. Following incubation, cells were washed three times with PBS to remove extracellular probes. Subsequently, intracellular ROS production was detected and quantitatively analyzed using a using a flow cytometry.

### 4.20 Mitotracker and MitoSOX Staining

For MitoTracker and MitoSOX staining, primary astrocytes or neurons in different groups were stained with MitoTracker Deep Red FM (C1032, Beyotime) or MitoSOX Red (HY‐D1055, MCE), respectively. Images were captured using a confocal fluorescence microscope or a fluorescence microscope (Leica, Germany). MitoSOX fluorescence intensity was quantitatively analyzed using ImageJ software.

### 4.21 Mitochondrial Membrane Potential Measurement

Evaluation of mitochondrial membrane potential was performed using the JC‐1 Assay Kit (C2006, Beyotime) following the manufacturer's instructions. The membrane potential was determined by calculating the fluorescence intensity ratio of aggregates to monomers.

### 4.22 Astrocyte Proliferation and Wound Healing

Primary astrocyte proliferation was assessed using an EdU‐594 cell proliferation assay kit (C0078L, Beyotime). During the assay, EdU working solution (10 µM) was added to the culture medium and incubated at 37°C for 2 h. Following incubation, cells were trypsinized and collected to prepare a single‐cell suspension. The cells were fixed with 4% paraformaldehyde for 15 min at room temperature and then washed with PBS containing 3% BSA. After permeabilization with 0.3% Triton X‐100 for 15 min, 500 µL of freshly prepared Click reaction cocktail was added to each tube and incubated for 30 min at room temperature in the dark. The percentage of EdU‐positive cells within the total cell population was analyzed by flow cytometry to quantitatively reflect the astrocyte proliferation rate. A scratch assay was used to test wound healing in astrocytes in vitro as we previously described [58].

### 4.23 Western Blot Analysis

Total proteins were extracted from cells and spinal cord tissues using radioimmunoprecipitation assay lysis buffer (P0013B, Beyotime). Protein concentrations were determined using a BCA assay kit (P0012, Beyotime). After denaturation at 100°C for 10 min and separation via electrophoresis, the proteins were transferred onto polyvinylidene fluoride membranes, which were subsequently blocked with 5% skim milk. The membranes were incubated overnight with primary antibodies at 4°C, washed with Tris‐buffered saline/Tween‐20, and then incubated with horseradish peroxidase‐conjugated secondary antibodies for 1 h at room temperature. Protein bands were visualized using enhanced chemiluminescence reagent (180–501, Tanon) and quantified using ImageJ software. The primary antibodies used were as follows: mouse anti‐CPT1A (66039‐1‐Ig, Proteintech), mouse anti‐SOX9 (67439‐1‐Ig, Proteintech), rabbit anti‐HMGCL (16898‐1‐AP, Proteintech), rabbit anti‐HMGCS2 (HPA027442, Sigma‐Aldrich), mouse/rabbit anti‐GFAP (GB12096, GB11096, Servicebio), rabbit anti‐NeuN (GB11138, Servicebio), rabbit anti‐IBA1 (GB114490, Servicebio), mouse anti‐β‐actin (66009‐1‐Ig, Proteintech), rabbit anti‐Kbhb (PTM‐1204RM, PTM Bio), rabbit anti‐Flag (80010‐1‐RR, Proteintech), rabbit anti‐HA (51064‐2‐AP, Proteintech), rabbit anti‐P300 (20695‐1‐AP, Proteintech), rabbit anti‐ATG5 (10181‐2‐Ig, Proteintech), rabbit anti‐Beclin 1 (11306‐1‐AP, Proteintech), rabbit anti‐LC3 (14600‐1‐AP, Proteintech), rabbit anti‐OPTN (10837‐1‐AP, Proteintech), rabbit anti‐histone H3 (PTM‐6621, PTM Bio), rabbit anti‐H3K9bhb (PTM‐1250RM, PTM Bio), mouse anti‐H3K18bhb (PTM‐1292, PTM Bio), rabbit anti‐H4K8bhb (PTM‐1253RM PTM Bio) and mouse anti‐H3K27bhb (PTM‐1293RM PTM Bio).

### 4.24 Co‐IP

Protein extraction was performed on astrocytes or HEK293T cells transfected with the specified plasmids. The resulting cell lysates were subjected to immunoprecipitation using the corresponding antibodies conjugated to magnetic beads, following the instructions of the Pierce Crosslink Magnetic IP/Co‐IP kit (88805, Thermo Fisher Scientific). Following immunoprecipitation, the samples were collected and washed five times with lysis buffer to remove any non‐specific binding. The immunoprecipitated proteins were then analyzed in subsequent Co‐IP experiments.

### 4.25 Kbhb Peptides Immunoaffinity Enrichment and LC‐MS/MS Analysis

To qualitatively identify Kbhb peptides, small tissue pieces were taken from the spinal cord of mice at 14 dpi. The tissue was washed with cold PBS and homogenized on ice with lysis buffer (8 M urea, 2 mM EDTA, 3 mM TSA, 50 mM NAM, 5 mM DTT, and 1% Protease Inhibitor Cocktail III). Residual debris was removed by centrifugation at 18 000 g for 3 min at 4°C. Peptides were fractionated into 23 fractions by high pH reverse‐phase HPLC using an Agilent 300 Extend C18 column (5 µm particles, 4.6 mm ID, 250 mm length) and vacuum‐dried. To enrich for Kbhb‐modified peptides, the peptides dissolved in NETN buffer (100 mM NaCl, 1 mM EDTA, 50 mM Tris‐HCl, 0.5% NP‐40, pH 8.0) were incubated with 30 µL pre‐washed anti‐pan‐Kbhb antibody beads (PTM‐1204, PTM Bio) at 4°C overnight with gentle shaking. The beads were washed four times with NETN buffer and twice with ddH2O. The bound peptides were eluted from the beads with 0.1% trifluoroacetic acid. The eluted peptides were vacuum‐dried and subjected to LC‐MS/MS analysis.

### 4.26 ATAC‐Seq

Mice were euthanized under anesthesia, and astrocytes were isolated from spinal cord tissues via MACS to prepare a single‐cell suspension. The samples were processed using the Hyperactive ATAC‐Seq Library Prep Kit for Illumina (TD711‐01, Vazyme) according to the manufacturer's instructions. Briefly, nuclei were extracted using lysis buffer and subsequently incubated with Tn5 transposase to cleave and simultaneously insert sequencing adapters into open chromatin regions. The resulting DNA fragments were purified and recovered, followed by library enrichment via PCR amplification. Post‐PCR, the libraries were purified using magnetic beads. Finally, after passing quality control, the libraries were sequenced on the Illumina platform.

### 4.27 CUT&Tag

Primary astrocytes were collected to prepare a single‐cell suspension as described above. The samples were processed using the Hyperactive Universal CUT&Tag Assay Kit for Illumina Pro (TD904‐01/02, Vazyme) according to the manufacturer's instructions. Briefly, the sorted cells were incubated with activated ConA beads at room temperature for 30 min to allow binding. The cell‐bead complexes were resuspended in antibody binding buffer containing Digitonin, and the specific primary antibody (H3K9bhb) was added for overnight incubation at 4°C. After removing the supernatant, the secondary antibody was added and incubated at room temperature for 1 h, followed by incubation with pAG‐Tn5 transposase at room temperature for 1 h after washing. Subsequently, digestion buffer was added and incubated at 37°C for 1 h for fragmentation. Finally, DNA fragments were extracted using DNA Extract Beads, followed by PCR amplification and purification. The final libraries were sequenced on the Illumina platform after passing quality control.

### 4.28 ATAC‐Seq and CUT&Tag Data Analysis

ATAC‐seq and CUT&Tag (H3K9bhb) libraries were prepared and sequenced. Raw reads were trimmed using fastp and aligned to the mouse reference genome (mm10) using Bowtie2 with default parameters. Duplicate reads were removed using Picard. Peak calling was performed for each sample independently using MACS2 with a q‐value threshold of < 0.05. Peaks from both conditions were merged into a unified peak set using BEDTools. Read counts within merged peak regions were quantified using featureCounts and normalized to RPKM (reads per kilobase per million mapped reads). The RPKM ratio (fold change, FC) between SCI and sham groups was calculated as a descriptive metric for differential signal, with FC ≥ 2 designated as signal‐enriched regions and FC ≤ 0.5 as signal‐depleted regions. Normalized signal tracks were generated using deepTools bamCoverage with RPKM normalization and visualized using the Integrative Genomics Viewer (IGV) and deepTools. Motif enrichment analysis of differentially accessible regions was performed using HOMER.

### 4.29 ChIP‐qPCR

ChIP‐qPCR analysis was performed using a ChIP Assay Kit (P2078, Beyotime) according to the manufacturer's protocol. Briefly, isolated primary astrocytes were crosslinked with 1% formaldehyde for 10 min at room temperature, followed by the addition of glycine solution to quench the reaction. Cells were collected and lysed, and the resuspended nuclei were sheared into chromatin fragments of 200–800 bp using a sonicator. Crosslinks between proteins and DNA were removed by adding NaCl and heating at 65°C for 4 h. Chromatin was then extracted with tris‐phenol and chloroform. The sheared chromatin was incubated with IgG or H3K9bhb antibodies overnight at 4°C with constant rotation. Precipitates were incubated with elution buffer at 60°C for 4 h. Finally, the DNA was purified using a DNA Purification Kit (D0033, Beyotime). H3K9bhb occupancy on *Acat2*, *Acadl*, *Acaa1a*, *Hmgcl*, *Mki67*, *Pcna*, *Myc*, and *Tgfb3* was examined through ChIP sequencing and qPCR analysis. The primer sequences used are listed in Table S2.

### 4.30 RT‐qPCR

Total RNA was extracted from mouse spinal cords and primary astrocytes using RNAiso Plus reagent (9108, Takara) following the manufacturer's protocol. After RNA purification and quantification, gDNA wiper Mix was added to the RNA samples in RNase‐free tubes and incubated at 42°C for 2 min to remove genomic DNA contamination. Subsequently, the reverse transcription master mix was added, and the reaction was performed at 50°C to synthesize cDNA, followed by a brief incubation at 85°C to inactivate the reverse transcriptase. The resulting cDNA was used for RT‐qPCR. Next, RT‐qPCR was performed using the TB Green Premix Ex Taq kit (RR420B, Takara) in an ABI 7900HT qPCR system (Applied Biosystems, Thermo Fisher). The relative mRNA expression of *Acat2, Acadl, Acaa1a, Hmgcl, Mki67, Pcna, Myc*, and *Tgfb3* was normalized to *Actb* and quantified using the 2^−ΔCT^ method. The primer sequences used are listed in Table S3.

### 4.31 Transfection Assay

Full‐length sequences of CPT1A, CPT1A mutation, P300, GCN5, PCAF, CBP, P62, NBR1, NDP52, Tollip and OPTN were PCR‐amplified and cloned into vector tagged with Flag or HA. Lipofectamine 3000 reagent (L3000001, Invitrogen) was used for transfection according to the manufacturer's instructions. Astrocytes were transfected with adenoviruses containing shRNA‐control, sh*p300*, sh*Atg5*, sh*Becn1*, and sh*Optn* (GenePharma, China). The shRNA sequences used are listed in Table S4.

### 4.32 TEM

Samples were first immersed overnight in chilled 2% glutaraldehyde (0.1 M cacodylate buffer, pH 7.4) at 4°C, then rinsed and post‐fixed with 1% osmium tetroxide (phosphate‐buffered) for 1 h. Subsequently, the specimens underwent dehydration through a graded acetone series before being infiltrated and embedded in EPON resin. Ultrathin sections were cut using a Leica EM UC7 microtome (Leica Microsystems) and stained with 3% uranyl acetate followed by lead citrate. High‐resolution imaging was performed on an FEI Tecnai electron microscope (Hillsboro, OR, USA). Quantitative analysis was performed across six randomly selected fields.

### 4.33 Statistical Analysis

All statistical analyses were performed using GraphPad Prism (version 9.5.1). Data are presented as the mean ± standard error of the mean (SEM). Differences between two groups were determined using a two‐tailed Student's *t*‐test. The relationship between two continuous variables was examined by Pearson correlation analysis. For multi‐factor comparisons, we performed either one‐way or two‐way ANOVA, with Bonferroni correction applied for post hoc testing. Statistical significance was set at *p* < 0.05, with results denoted as follows: *p < 0.05, **p < 0.01, ***p < 0.001; n.s. indicates not significant. Sample sizes are provided in the corresponding figure legends. For all behavioral and immunohistochemical assessments, investigators were blinded to group allocation during data collection and analysis.

## Author Contributions

**Dishui Pan**: Formal analysis, Methodology, Investigation, Funding acquisition, Writing – original draft; **Haoming Shu**: Methodology, Investigation, Visualization; **Rui Gao**: Methodology, Investigation, Visualization; **Yilong Ren**: Methodology, Investigation, Visualization; **Wu Ye**: Formal analysis, Investigation; **Xuhui Ge**: Investigation, Validation; **Yao Gu**: Investigation, Validation; **Yufeng Zhu**: Investigation, Validation; **Xiaokun Wang**: Investigation, Validation; **Honglin Teng**: Writing – review & editing; **Xuhui Zhou**: Writing – review & editing; **Wei Liu**: Conceptualization, Writing – original draft, Writing – review & editing, Supervision; **Yang Gao**: Conceptualization, Writing – review & editing, Supervision; **Jianquan Zhao**: Conceptualization, Writing – review & editing, Supervision; **Weihua Cai**: Conceptualization, Funding acquisition, Project administration, Writing – review & editing, Supervision.

## Conflicts of Interest

The authors declare no conflicts of interest.

## Supporting information




**Supporting File**: advs78393‐sup‐0001‐SuppMat.pdf.

## Data Availability

The data that support the findings of this study are available from the corresponding author upon reasonable request.
