# Supplementary material for: Astrocytic Ketogenesis Protects Central Nervous System Injury via CPT1A Stabilization and Epigenetic Regulation
Source: Adv Sci (Weinh). 2026 Oct 20:e78393. Online ahead of print. doi: 10.1002/advs.78393 (PMC13683976; doi:10.1002/advs.78393)
Supplement: Supplementary file 1 [file ADVS-9999-e78393-s001.pdf]

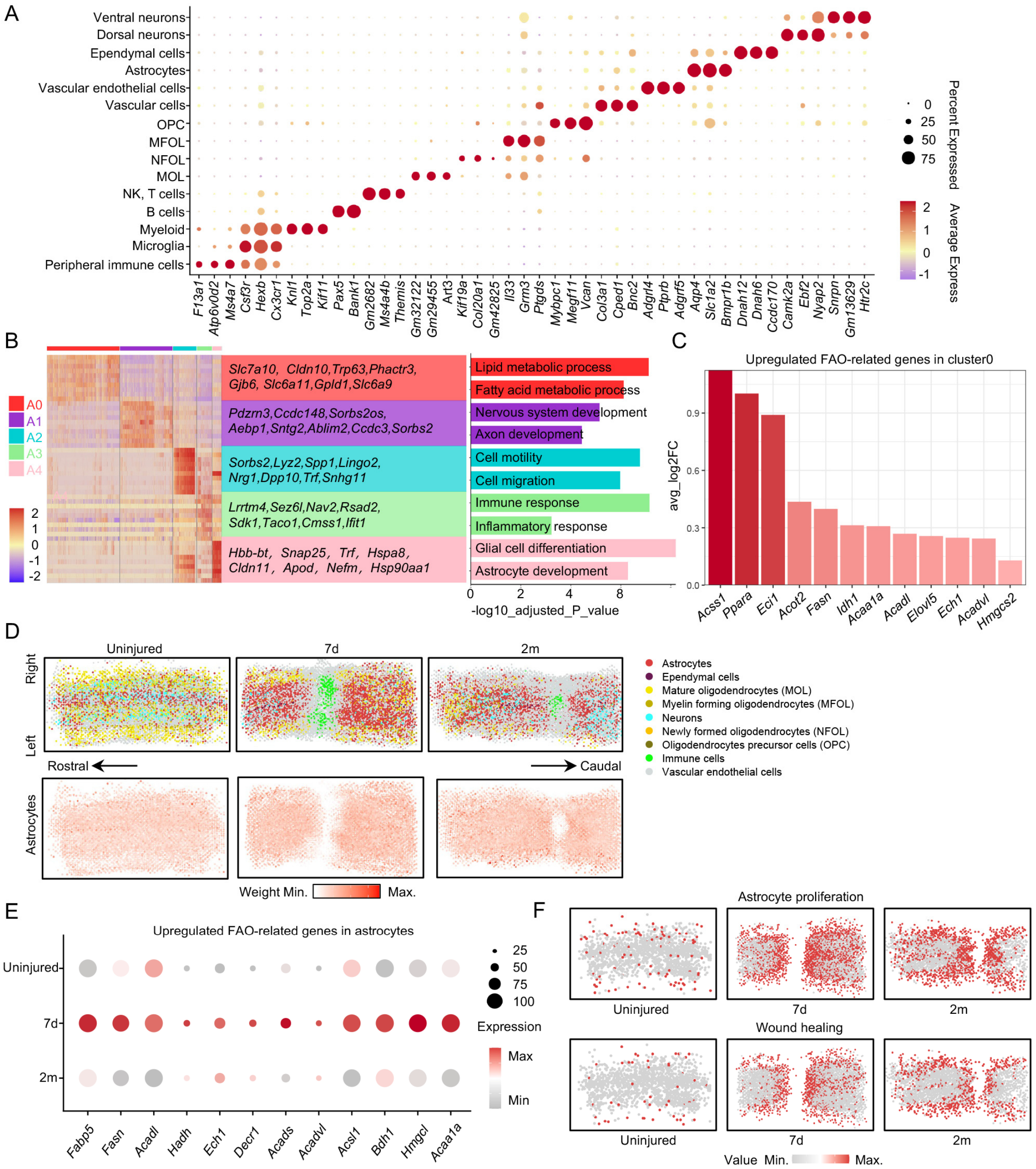

Figure S1. Astrocytes with upregulated lipid and ketone body metabolism spatially overlapped with proliferating astrocytes after SCI. (A) The dot plot shows the marker genes used for cellular annotation in Figure 1A. (B) Heatmap of marker genes used to re-cluster astrocytes and GO of biological process analysis using the top 50 marker genes. (C) Bar graph showing the average log_2FC of FAO-related genes in Cluster 0. (D) Cell-type deconvolution of spatial barcodes in the 2D spatial transcriptomic atlas, and the isolated astrocyte populations. (E) Dot plot showing the expression of FAO-related genes in astrocytes at the indicated time points from ST data. (F) Spatial distribution of gene set scores for astrocyte proliferation and wound healing metabolism across spatial barcodes.

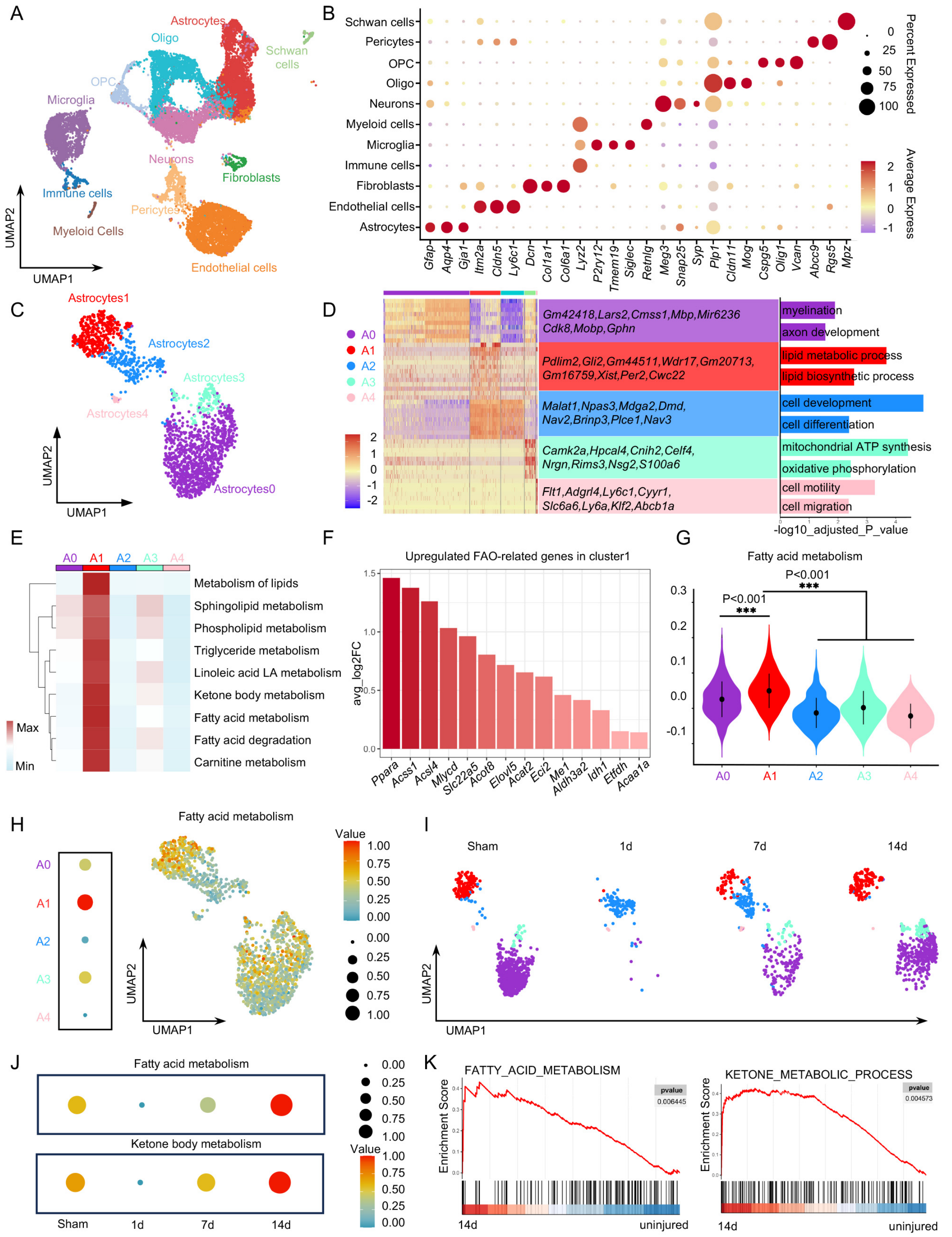

Figure S2. Astrocytic lipid and ketone metabolism is upregulated after SCI. (A) UMAP plot showing clusters of spinal cord cells after SCI. (B) Dot plot showing marker genes used for cell annotation in A. (C) UMAP plot showing populations of re-clustered astrocytes. (D) Heatmap of marker genes used to re-cluster astrocytes and GO of biological process analysis using the top 50 marker genes. (E) KEGG enrichment analysis of lipid metabolism-related pathways among subclusters showing enrichment in the A1 subcluster.

(F) Bar graph showing the average log_2FC of FAO-related genes in Cluster 1. (G) Fatty acid metabolism gene set scores across astrocyte subclusters. (H) UMAP plot and dot plot showing fatty acid metabolic pathways in each cluster of astrocytes. (I) UMAP plots of astrocytes at the indicated times. (J) Dot plot shows the lipid metabolic pathway and ketone body metabolic pathway in astrocytes at the indicated time points. (K) GSEA plot of fatty acid metabolism and ketone body metabolism in astrocytes at 14 dpi. Data were analyzed using one-way ANOVA followed by post hoc Bonferroni correction (G).

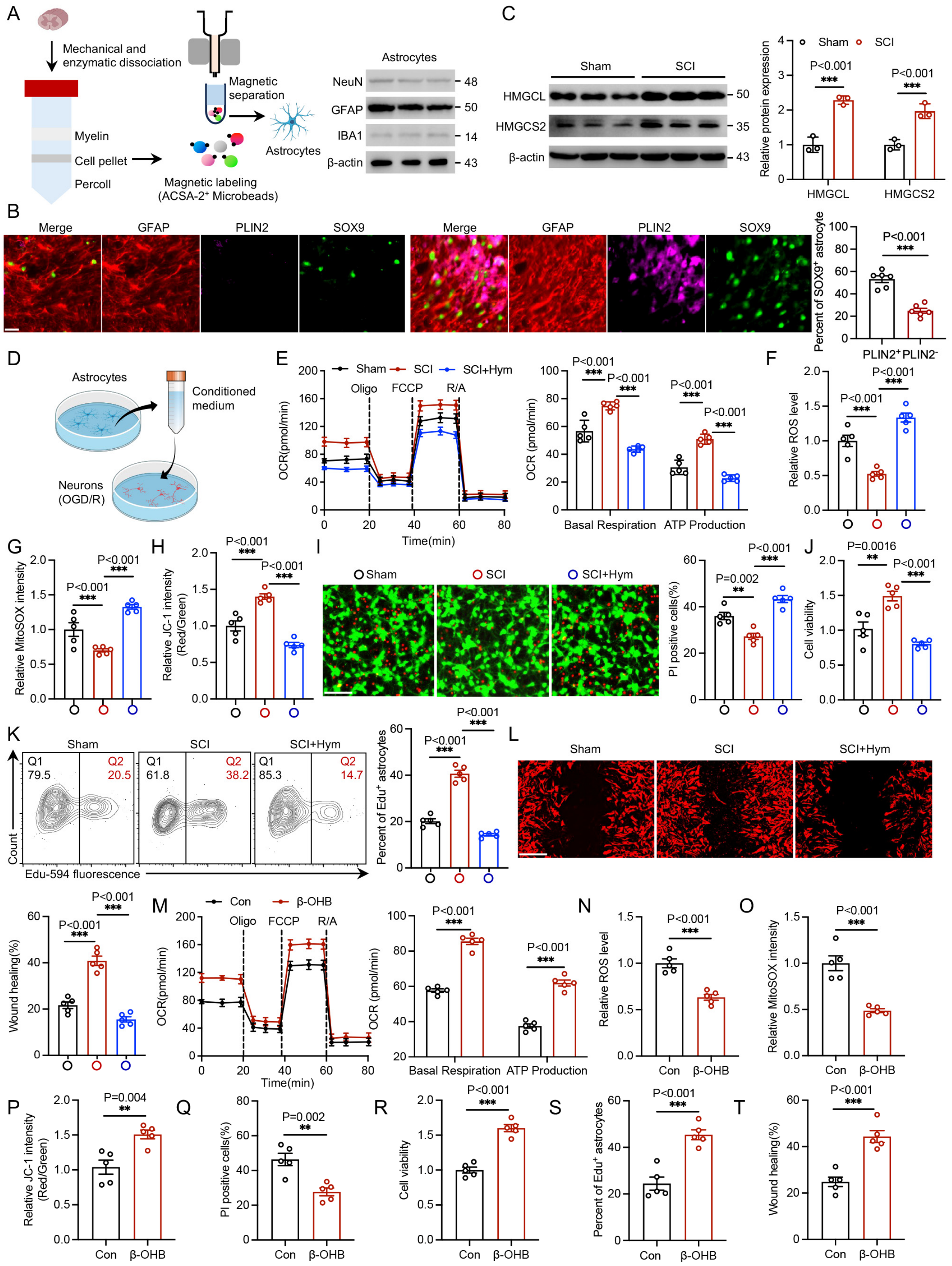

Figure S3. Upregulated lipid and ketone metabolism in astrocytes protects neurons, promotes astrocyte proliferation, and facilitates wound healing. (A) Western blot analysis of astrocytes immunomagnetically isolated from the mouse spinal cord. (B) Representative images of PLIN2, SOX9, and GFAP co-staining in the spinal cord of SCI mice at 14 dpi (n = 6 per group). Scale bar = 20 μm. (C) Western blot analysis of HMGCL and HMGCS2 protein levels in astrocytes isolated from the spinal cords of sham and SCI

mice (n = 3 per group). (D) Schematic diagram of the conditioned medium transfer strategy. (E) OCR analysis of primary neurons cultured with conditioned media from different groups of astrocytes, with quantification of basal respiration and ATP production (n = 5 per group). (F-H) Quantitative analysis of ROS (F), MitoSOX (G), JC-1 (H) levels in primary neurons from different experimental groups (n = 5 per group). (I) Representative images and quantification of neuronal death in each group using PI and Calcein-AM staining (n = 5 per group). Scale bar = 50 μm. (J) Cell viability of primary neurons in each group (n = 5 per group). (K) Proliferation of astrocytes in each group (n = 5 per group). (L) Representative images and quantitative analysis of wound healing in astrocytes from different groups following a scratch assay (n = 5 per group). Scale bar = 50 μm. (M) OCR analysis and quantification of basal respiration and ATP production in primary neurons following exogenous β-OHB supplementation. (N-P) Quantitative analysis of ROS (N), MitoSOX (O), JC-1 (P) levels in primary neurons after β-OHB supplementation (n = 5 per group). (Q) Quantification of neuronal death after β-OHB supplementation (n = 5 per group). (R) Cell viability of primary neurons after β-OHB supplementation (n = 5 per group). (S) Proliferation of astrocytes after β-OHB supplementation (n = 5 per group). (T) Quantitative analysis of wound healing in astrocytes following a scratch assay after β-OHB supplementation (n = 5 per group). Data were analyzed using two-tailed unpaired Student's *t*-test (B, C, M-T) and one-way ANOVA followed by post hoc Bonferroni correction (E-L).

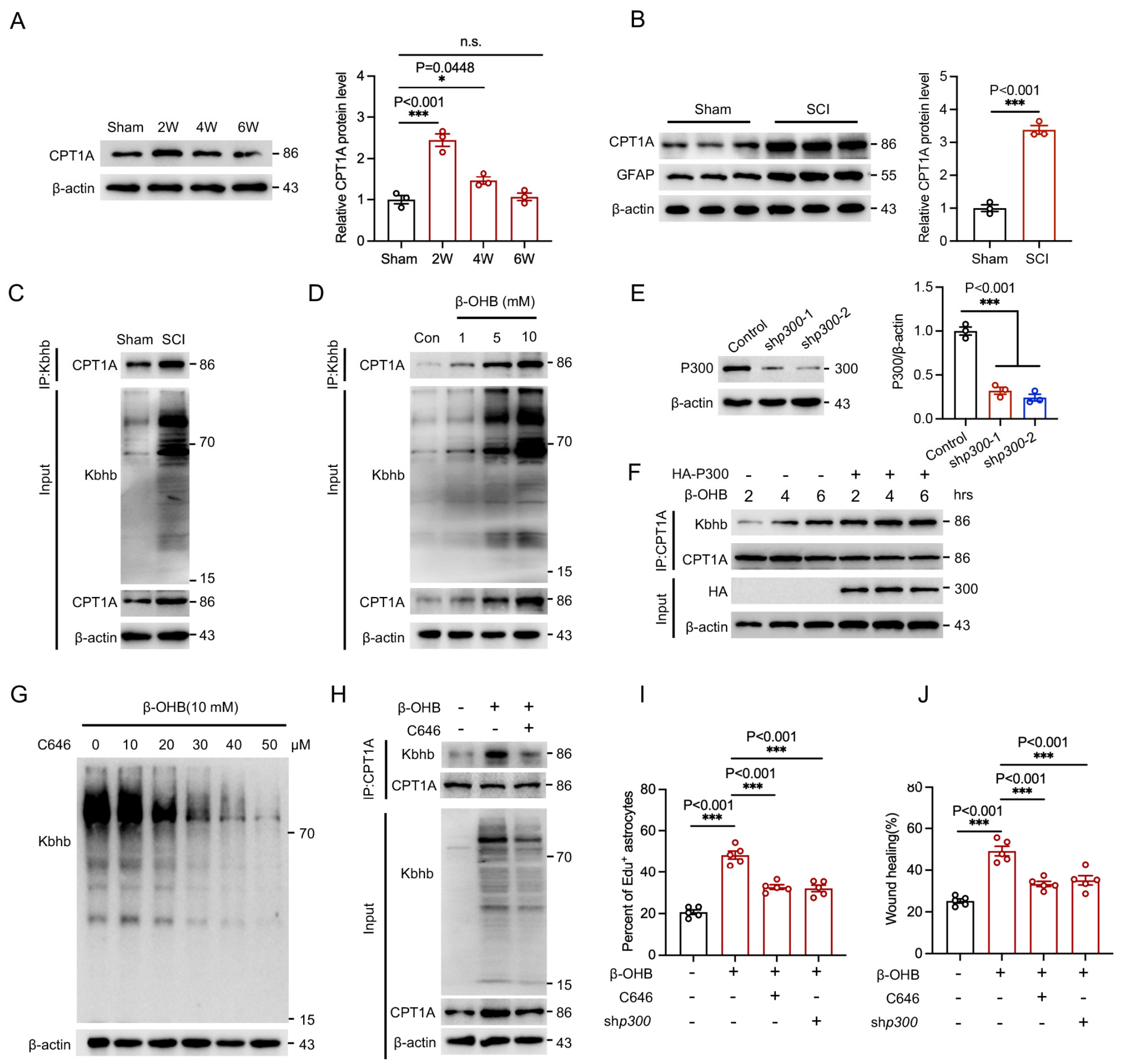

Figure S4. β-OHB promotes CPT1A Kbhb modification at lysine 344 via P300. (A) Spinal cord CPT1A protein levels at the specified time points after SCI. (B) CPT1A protein levels in isolated astrocytes from sham and SCI groups. (C) Spinal cord tissue protein lysates were immunoprecipitated with an anti-Kbhb antibody followed by immunoblotting with an anti-CPT1A antibody. (D) Primary astrocytes treated with a concentration gradient of β-OHB were subjected to immunoprecipitation with an anti-Kbhb antibody followed by immunoblotting with an anti-CPT1A antibody. (E) The knockdown efficiency of *p300* in astrocytes was assessed by immunoblotting. (F) Astrocytes transfected with or without HA-P300 were treated with 10 mM β-OHB for various durations. Cell lysates were then subjected to immunoprecipitation with an anti-CPT1A antibody followed by detection of the Kbhb levels of CPT1A. (G) Primary astrocytes were treated with 10 mM β-OHB and the indicated concentrations of C646 for 24 hours, and Kbhb levels were determined by

immunoblotting with an anti-Kbhb antibody. (H) Astrocytes were treated with 10 mM β-OHB for 24 hours in the presence or absence of C646. Cell lysates were then subjected to immunoprecipitation with an anti-CPT1A antibody, followed by detection of the Kbhb levels of CPT1A. (I, J) Quantitative analysis of the proliferation ratio (I) and wound healing (J) in astrocytes from different groups (n = 5 per group). Data were analyzed using two-tailed unpaired Student's *t*-test (B) and one-way ANOVA followed by post hoc Bonferroni correction (A, E, I, J).

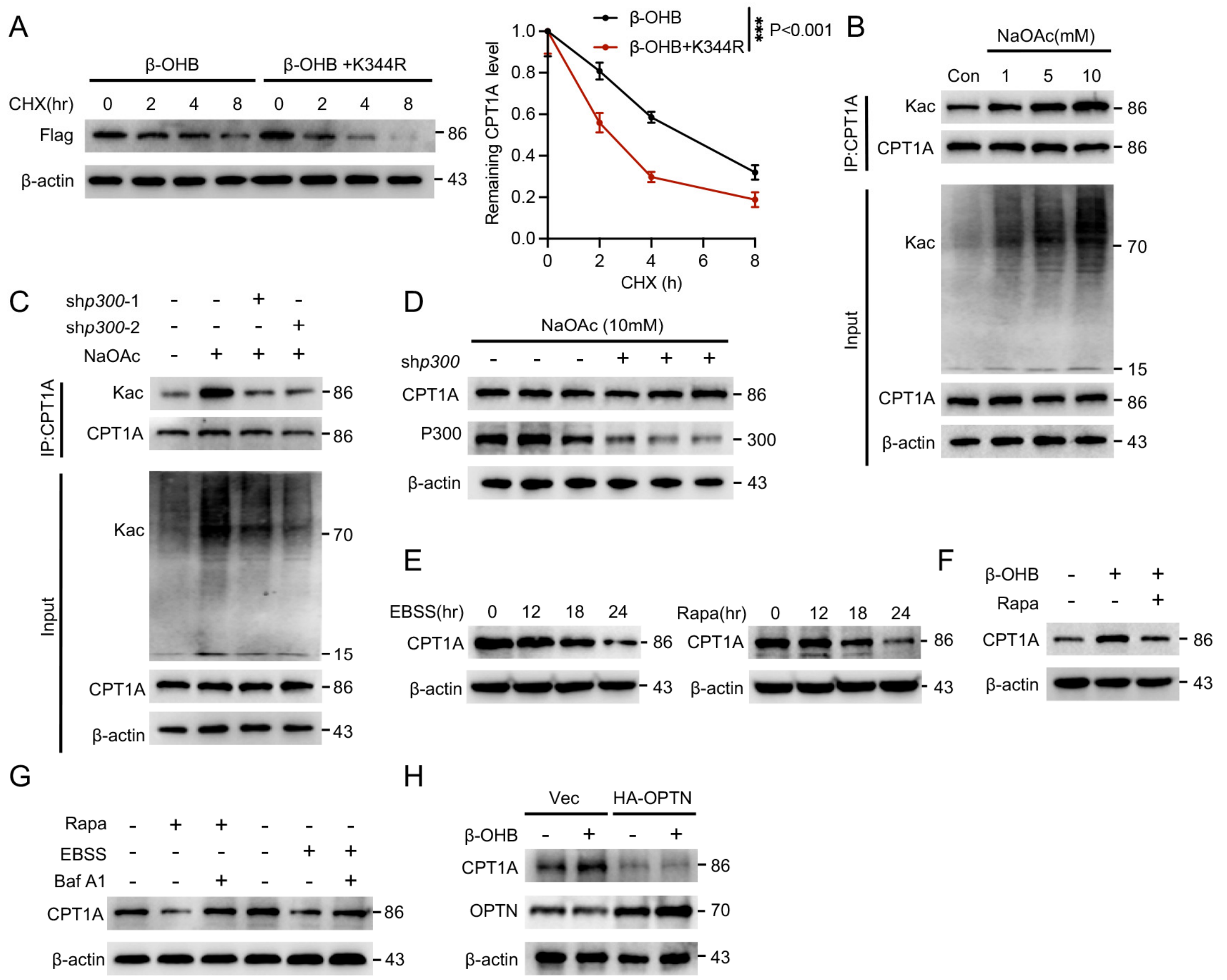

Figure S5. Kbhb inhibits CPT1A selective autophagic degradation via the cargo receptor OPTN. (A) HEK293T cells transfected with Flag-CPT1A (WT or K344R) were treated with 10 mM β-OHB for 24 hours, followed by immunoblotting to detect Flag-CPT1A protein levels. (B) Primary astrocytes treated with a concentration gradient of NaOAc (sodium acetate) were subjected to immunoprecipitation with an anti-CPT1A antibody, followed by detection of CPT1A Kac levels using an anti-Kac antibody. (C) Astrocytes transfected with or without sh*p300* were treated with 10 mM NaOAc for 24 hours, followed by immunoblotting to detect CPT1A Kac levels. (D) Primary astrocytes transfected with or without sh*p300* were treated with 10 mM NaOAc for 24 hours, followed by immunoblotting to assess CPT1A protein levels. (E) Primary astrocytes were treated with rapamycin (250 nM) or EBSS for indicated time points, and the proteins were harvested for immunoblot analysis. (F) Primary astrocytes were treated with β-OHB in the presence or absence of rapamycin (250 nM) for 24 hours. Cell lysates were then collected for immunoblotting analysis. (G) Primary astrocytes were treated with rapamycin (250 nM) or cultured in EBSS, in the presence or absence of Baf A1 (0.2 μM). Cell lysates were then harvested for

immunoblotting analysis. (H) Astrocytes transfected with or without HA-OPTN were treated with or without β-OHB, followed by immunoblotting to detect CPT1A protein levels. Data were analyzed by two-way ANOVA followed by post hoc Bonferroni correction (A).

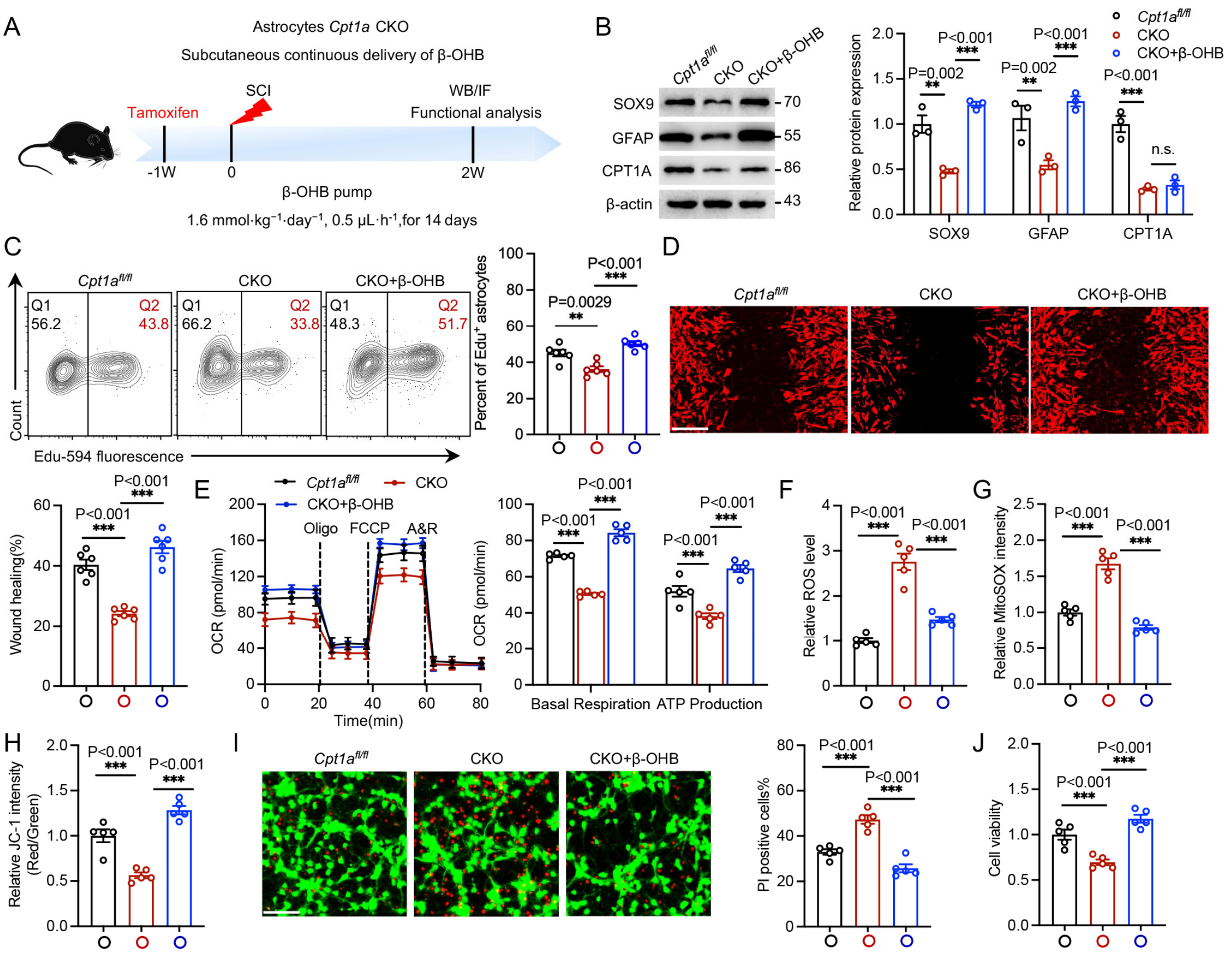

Figure S6. Astrocyte-specific deletion of *Cpt1a* impaired astrocyte proliferation and migration and promoted neuronal death, both of which were rescued by exogenous β-OHB. (A) Schematic showing the conditional astrocyte-specific gene knockout strategy, β-OHB administration regimen, and functional or pathological analyses performed at specific time points after SCI. (B) Astrocytes from different groups were sorted, and the protein levels of SOX9, GFAP, and CPT1A were detected by immunoblotting (n = 3 per group). (C) Proliferation of astrocytes in each group (n = 6 per group). (D) Representative images and quantitative analysis of wound healing in astrocytes from different groups following a scratch assay (n = 6 per group). Scale bar = 50 μm. (E) OCR analysis of primary neurons cultured with conditioned media from different groups of astrocytes, with quantification of basal respiration and ATP production (n = 5 per group). (F-H) Quantitative analysis of ROS (F), MitoSOX (G), and JC-1 (H) levels in primary neurons from different experimental groups (n = 5 per group). (I) Representative images and quantification of neuronal death in each group using PI and Calcein-AM staining (n = 5 per group). Scale bar = 50 μm. (J) Cell viability of primary neurons in each group (n = 5 per group). Data were analyzed using one-way ANOVA followed by post hoc Bonferroni correction (B-J).

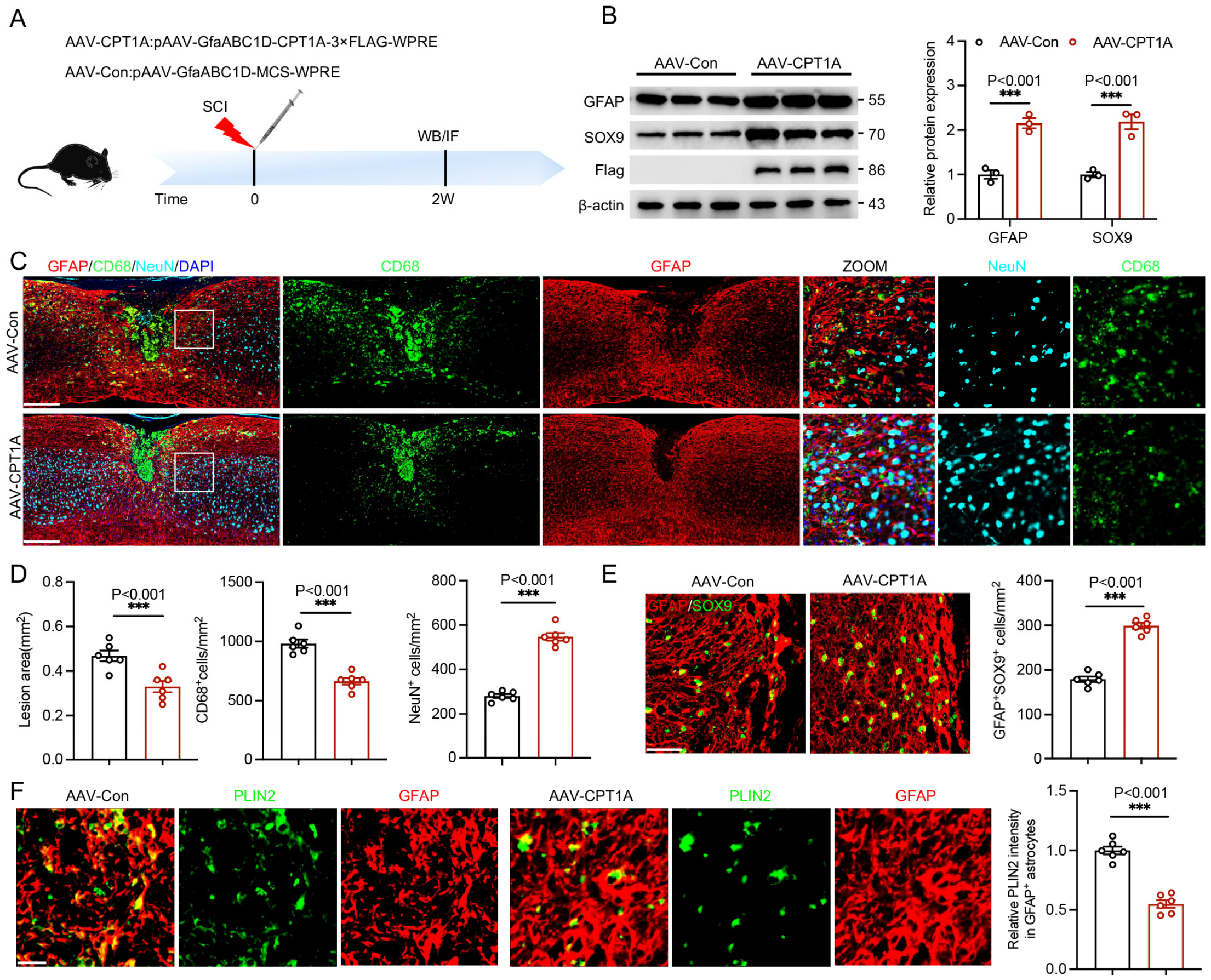

Figure S7. Overexpression of CPT1A in astrocytes enhanced tissue repair after SCI. (A) Schematic showing the gene overexpression strategy and analyses performed at specific time points after SCI. (B) Changes in GFAP and SOX9 protein levels in AAV-Con and AAV-CPT1A groups of mice at 14 dpi (n = 3 per group). (C, D) Representative images of CD68, GFAP, and NeuN co-staining in the spinal cord of mice at 14 dpi for the specified groups, along with quantification of lesion area, $CD68^+$ macrophages/microglia, and neurons (n = 6 per group). Scale bar = 500 μm. (E) Double staining of GFAP and SOX9 in the spinal cord and quantification of $GFAP^+SOX9^+$ cells at 14 dpi (n = 6 per group). Scale bar = 50 μm. (F) Double staining of GFAP and PLIN2 at 14 dpi and quantification of PLIN2 fluorescence intensity in $GFAP^+$ cells (n = 6 per group). Scale bar = 20 μm. Data were analyzed using a two-tailed unpaired Student's *t*-test (B, D-F).

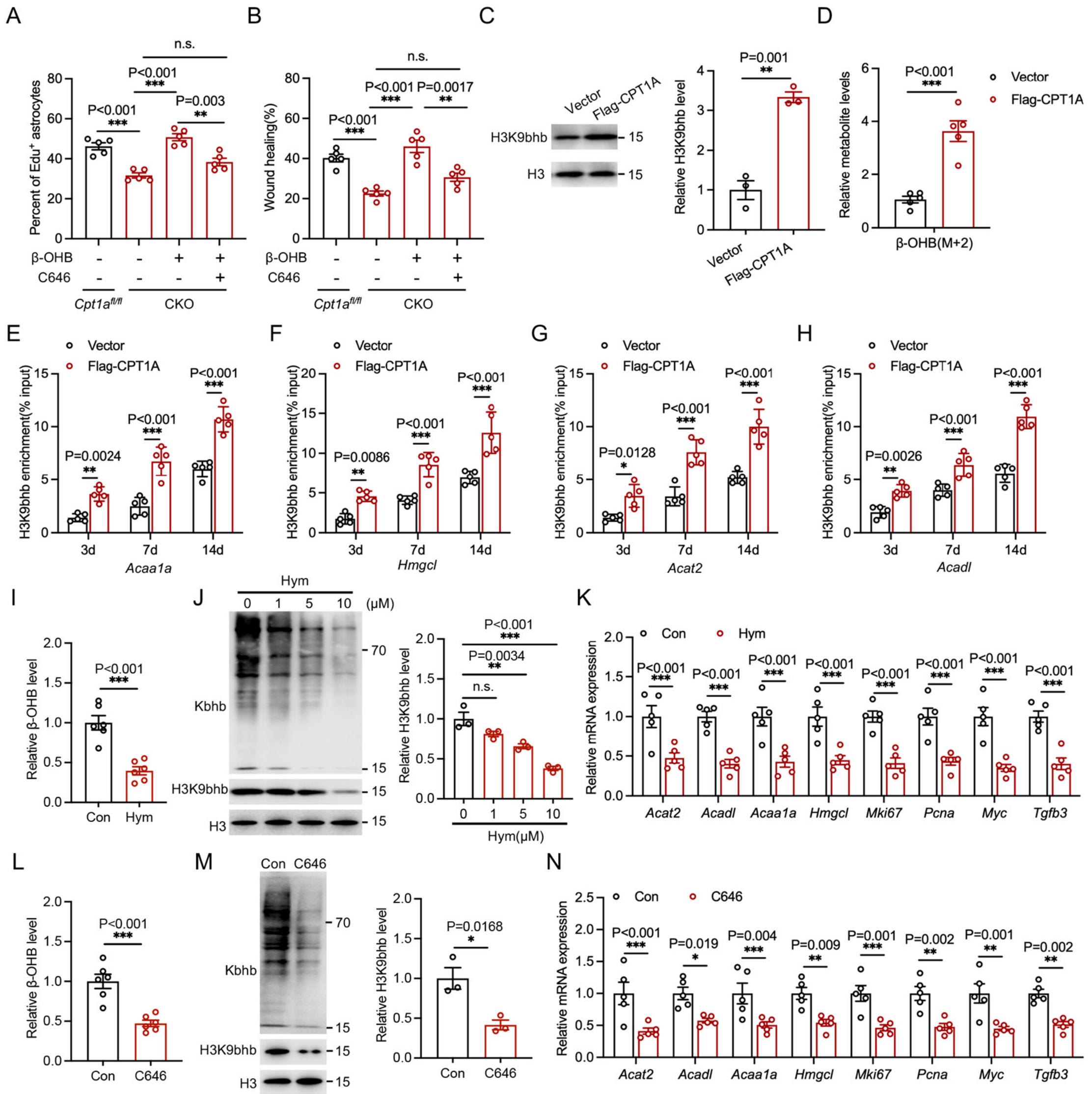

Figure S8. Astrocytic ketogenesis promotes wound repair and lipid metabolic reprogramming, forming a positive feedback loop via H3K9bhb-dependent epigenetic remodeling. (A, B) Quantitative analysis of the proliferation ratio (A) and wound healing (B) in astrocytes from different groups (n = 5 per group). (C) Immunoblotting analysis of H3K9bhb levels in astrocytes from different groups, along with quantification of H3K9bhb protein levels (n = 3 per group). (D) Relative abundance of metabolites in astrocytes of the indicated groups (n = 5 per group). (E-H) ChIP-qPCR analysis of the promoters of the indicated genes using anti-H3K9bhb antibody in control and CPT1A-overexpressing astrocytes (3, 7, and 14 dpi; n = 5 per group). (I) β-OHB levels in astrocytes with or without Hym treatment (n = 6 per group). (J) Immunoblotting analysis of Kbhb and H3K9bhb levels in astrocytes treated with a concentration gradient of Hym, along with quantification of H3K9bhb protein levels (n = 3 per group). (K) RT-qPCR analysis of mRNA

levels of relevant genes in Hym (10 μM)-treated or control astrocytes (n = 5 per group). (L) β-OHB levels in astrocytes with or without C646 treatment (n = 6 per group). (M) Immunoblotting analysis of Kbhb and H3K9bhb levels in astrocytes with or without C646 treatment, along with quantification of H3K9bhb protein levels (n = 3 per group). (N) RT-qPCR analysis of mRNA levels of relevant genes in C646 (50 μM)-treated or control astrocytes (n = 5 per group). Data were analyzed using a two-tailed unpaired Student's *t*-test (C-I, K-N) and one-way ANOVA followed by post hoc Bonferroni correction (A, B, J).

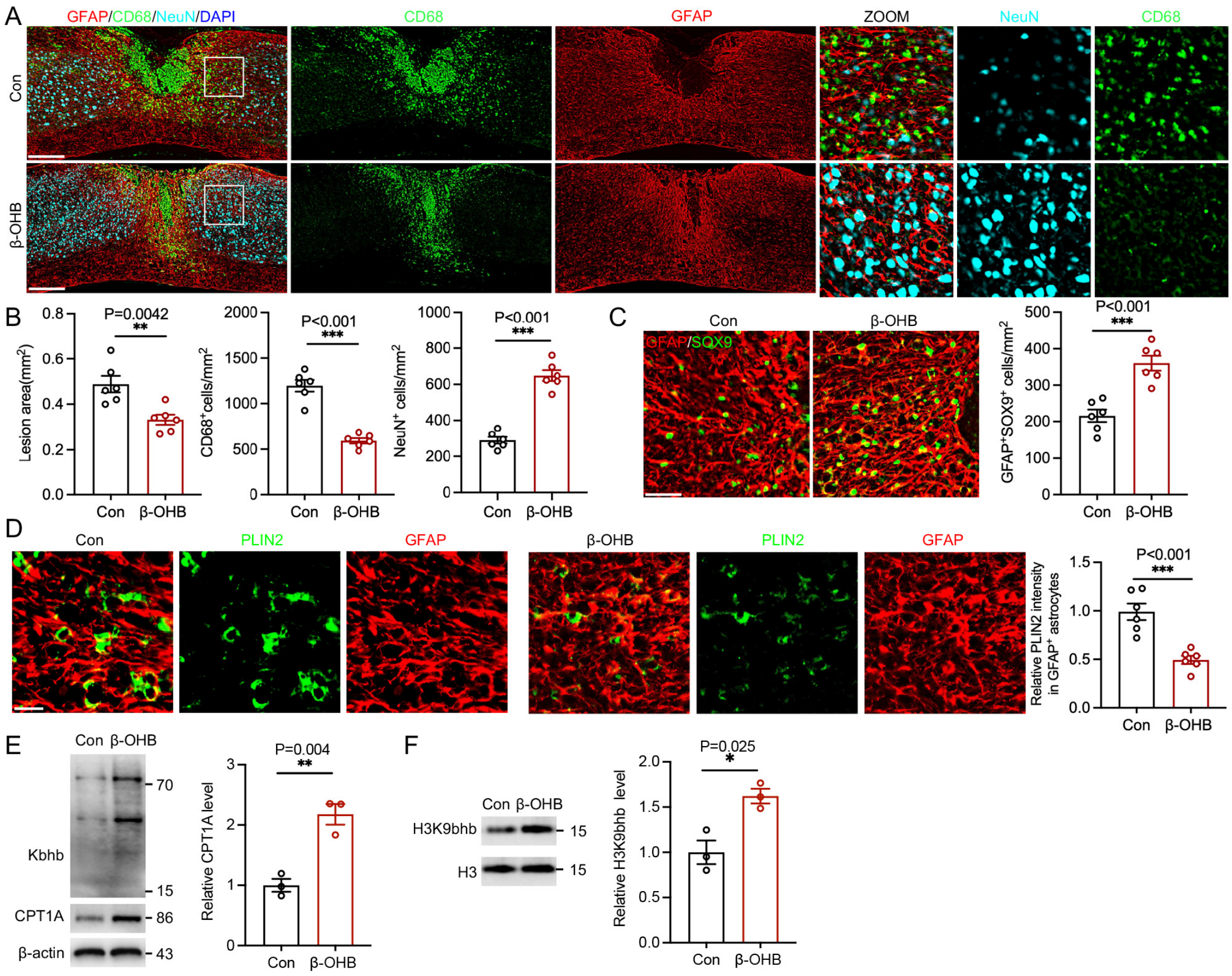

Figure S9. Exogenous β-OHB promotes tissue repair after spinal cord injury. (A, B) Representative images of CD68, GFAP, and NeuN co-staining in the spinal cord of mice at 14 dpi for the specified groups, along with quantification of lesion area, CD68⁺ macrophages/microglia, and neurons (n = 6 per group). Scale bar = 500 μm. (C) Double staining of GFAP and SOX9 in the spinal cord and quantification of GFAP⁺SOX9⁺ cells at 14 dpi (n = 6 per group). Scale bar = 50 μm. (D) Double staining of GFAP and PLIN2 at 14 dpi and quantification of PLIN2 fluorescence intensity in GFAP⁺ cells (n = 6 per group). (E) Immunoblotting analysis of Kbhb and CPT1A levels in astrocytes with or without β-OHB treatment (0.5 mM), along with quantification of CPT1A protein levels (n = 3 per group). (F) Immunoblotting analysis of H3K9bhb levels in astrocytes with or without β-OHB treatment (0.5 mM), along with quantification of H3K9bhb protein levels (n = 3 per group). Data were analyzed using a two-tailed unpaired Student's *t*-test (B-F).

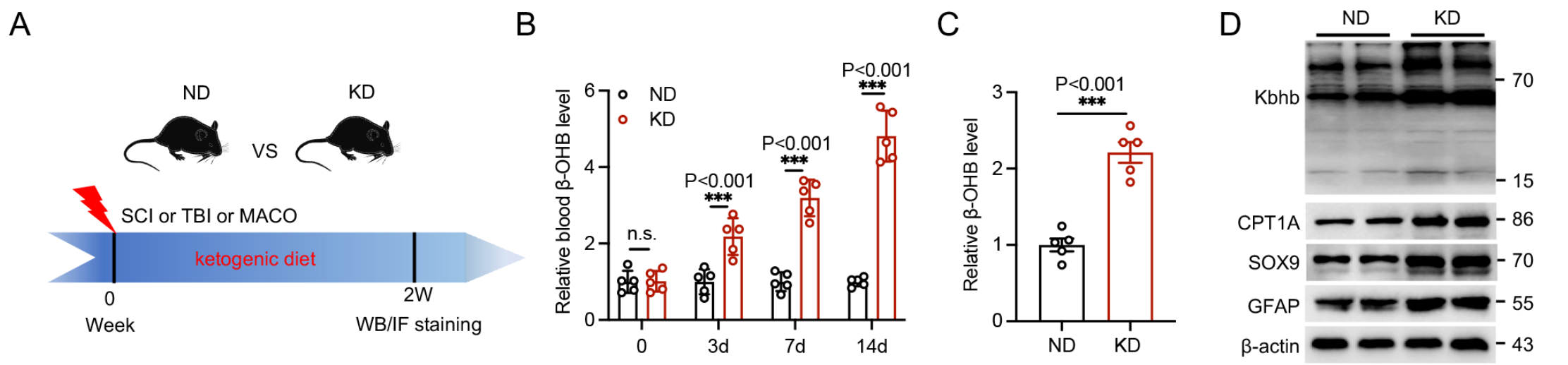

Figure S10. The ketogenic diet promotes tissue repair after CNS injury. (A) Multiple CNS injury models and KD strategy. (B) Peripheral blood β-OHB levels in mice at different time points on a KD in a SCI model (n = 5 per group). (C) Relative levels of spinal cord β-OHB in ND and KD mice (n = 5 per group). (D) Changes in Kbhb, CPT1A, SOX9, and GFAP protein levels in the ND and KD groups of mice at 14 dpi. Data were analyzed using a two-tailed unpaired Student's *t*-test (B, C).

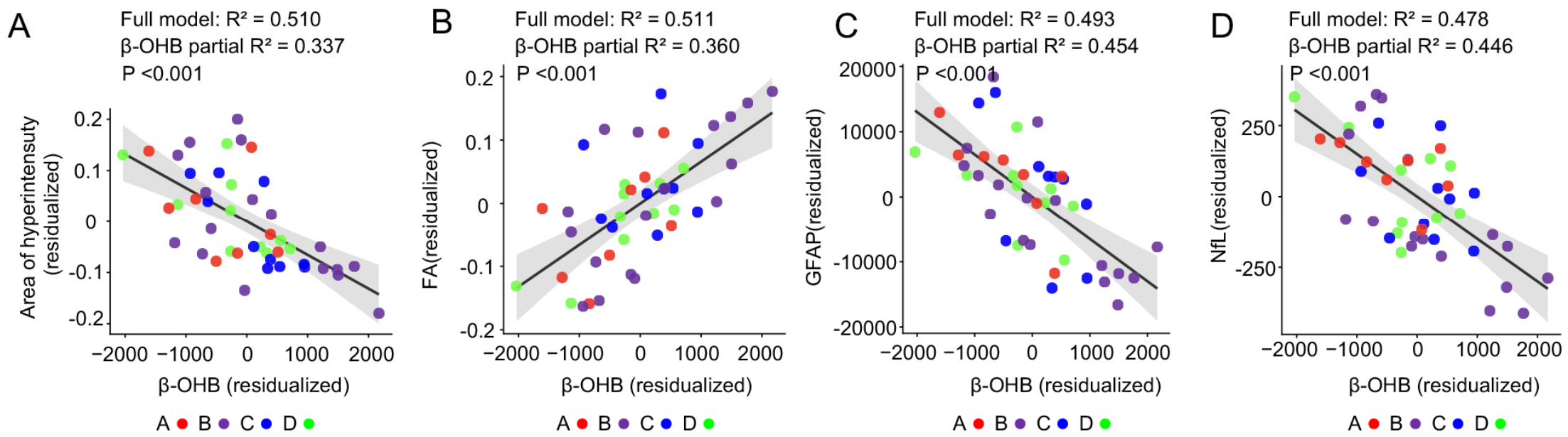

Figure S11. Multivariable-adjusted associations between CSF β-OHB and injury severity and biomarker outcomes. (A) Relationship between CSF β-OHB and T2 hyperintensity area. (B) Relationship between CSF β-OHB and fractional anisotropy at the lesion epicenter. (C) Relationship between CSF β-OHB and GFAP. (D) Relationship between CSF β-OHB and NF-L. All panels display partial regression results adjusted for ASIA grade, neurological level, age, sex, and injury-to-sampling interval. Solid lines represent linear fits, and shaded areas denote 95% confidence intervals. *P* values were derived from multivariable linear regression analysis.

Table S1. Detailed information of the recruited SCI patients and control subjects.

	Control	SCI	*P* value
Patient, n	20	45	
Age, y, mean (SD)	52.3 (15.93)	47.4 (12.37)	0.192
Gender			
male, n (%)	8 (40)	23 (51.1)	0.408
female, n (%)	12 (60)	22 (48.9)	
Time from injury to admission, h, mean (SD)	n.a.	18.5 (11.9)	
ASIA grade at admission, n (%)			
A	n.a.	8 (17.8)	
B	n.a.	17 (37.8)	
C	n.a.	10 (22.2)	
D	n.a.	10 (22.2)	
1-year neurologic outcome in ASIA-A/B patients			
ASIA grade improvement, n (%)	n.a.	8 (32.0)	
No ASIA grade improvement, n (%)	n.a.	17 (68.0)	

Table S2. The primers used in ChIP-qPCR analysis.

Gene	Sequence
Acat2-F	5'-GCAAAGGCGATGGTGACTTC-3'
Acat2-R	5'-TCGCTGATTGGTCTGTTCGG-3'
Acadl-F	5'-CCCACCACAGCATTTTGTCA-3'
Acadl-R	5'-ACTTCACCTCAACTCACCCCT-3'
Acaa1a-F	5'-CAAGGCAGGTTGTCACGCTA-3'
Acaa1a-R	5'-CAGCTATCAGACACCCCAGC-3'
Hmgcl-F	5'-CGTAGGTACCCCAGGACTCA-3'
Hmgcl-R	5'-CGCACGGCTTTCATTACCTG-3'
Mki67-F	5'-AGCTCATTAGCTCTGAACATCCA-3'
Mki67-R	5'-TGCGTTCTGTTCTGGGACTC-3'
Pcna-F	5'-CCCAAACTCTCTCAGGGGTG-3'
Pcna-R	5'-AAGGACACCCCGCTCAAAAA-3'
Myc-F	5'-TCTCTCCTTCCTCGGACTCG-3'
Myc-R	5'-GGGTAGCTTACCAGAGTCGC-3'
Tgfb3-F	5'-TCTCTCGCTGCACGGTTTTA-3'
Tgfb3-R	5'-CGTCGAACCAGAGGACTCAC-3'

Table S3. The primers used in RT-qPCR analysis.

Gene	Sequence
Cpt1a-F	5'-GGCATAAACGCAGAGCATTCCTG-3'
Cpt1a-R	5'-CAGTGTCCATCCTCTGAGTAGC-3'
Acat2-F	5'-GAGATTGTGCCAGTGCTGGTGT-3'
Acat2-R	5'-GTGACAGTTCCTGTCCCATCAG-3'
Acadl-F	5'-CCATGGCAAAATACTGGGCA-3'
Acadl-R	5'-GCAATCGGGTACTCCCACAT-3'
Acaa1a-F	5'-AATGTTCTTGAGCCAGGAGCC-3'
Acaa1a-R	5'-ACATGGACTCCACCCAGCAAT -3'
Hmgcl-F	5'- GCACTTTGCCAAAGCAGGTGAAG-3'
Hmgcl-R	5'-CGGAAAGCATGTCGATCAGCCT-3'
Mki67-F	5'-GAGGAGAAACGCCAACCAAGAG-3'
Mki67-R	5'-TTTGTCCTCGGTGGCGTTATCC-3'
Pcna-F	5'-AGCAACTTGGAATCCCAGAACA-3'
Pcna-R	5'-AATTCACCCGACGGCATCTT-3'
Myc-F	5'-TCGCTGCTGTCCTCCGAGTCC-3'
Myc-R	5'-GGTTTGCCTCTTCTCCACAGAC-3'
Tgfb3-F	5'-TTACTGCTTCCGCAACCTGG-3'
Tgfb3-R	5'-AGGTTCGTGGACCCATTTCC-3'
Actb-F	5'-GAGCTGCGTTTTACACCCT -3'
Actb-R	5'-GCCTTCACCGTTCCAGTTTT-3'

Table S4. The shRNA sequences.

Names	Species	Sequence
sh*p300*	Mus musculus	5'-CCCTGGATTAAGTTTGATAAA-3'
sh*Atg5*	Mus musculus	5'-GCCAAGTATCTGTCTATGATA-3'
sh*Becn1*	Mus musculus	5'-CCACATGTTTACAATACCAAA-3'
sh*Optn*	Mus musculus	5'-GAAGTCACAAAGAGGAATCTA-3'
